# Vinylation of *N*-Heteroarenes through Addition/Elimination Reactions of Vinyl Selenones

**DOI:** 10.3390/molecules28166026

**Published:** 2023-08-12

**Authors:** Martina Palomba, Italo Franco Coelho Dias, Michelangelo Cocchioni, Francesca Marini, Claudio Santi, Luana Bagnoli

**Affiliations:** Group of Catalysis, Synthesis and Organic Green Chemistry, Department of Pharmaceutical Sciences, University of Perugia, Via del Liceo, 1, 06123 Perugia, Italy

**Keywords:** vinyl selenones, azoles, addition/elimination reaction, domino process

## Abstract

A new protocol for the synthesis of *N*-vinyl azoles using vinyl selenones and azoles in the presence of potassium hydroxide was developed. This reaction proceeded under mild and transition metal-free conditions through an addition/elimination cascade process. Both aromatic and aliphatic vinyl selenones and various mono-, bi- and tri-cyclic azoles can be tolerated and give terminal *N*-vinyl azoles in moderate to high yields. A plausible mechanism is also proposed.

## 1. Introduction

*N*-vinyl azoles are common structural motifs of natural products, agrochemicals and pharmaceuticals and occupy an important place in heterocyclic chemistry, representing useful building blocks in organic synthesis and in material science [1,2]. *N*-vinyl imidazoles display antifungal activity [3], and diverse *N*-vinyl azoles are incorporated in structures of medical interest [4,5]. *N*-vinyl indole derivatives are useful intermediates for alkaloid synthesis [6,7,8]. They are also highly reactive monomers that generate polymeric materials with various properties. In particular, poly(*N*-vinyl indoles) are used as semiconductors and photosensitive materials [9,10] and the poly(*N*-vinyl carbazoles) are extensively studied photoconductive polymers with several applications such as light emitting diodes, capacitors or memory devices [11].

**Scheme 1:**
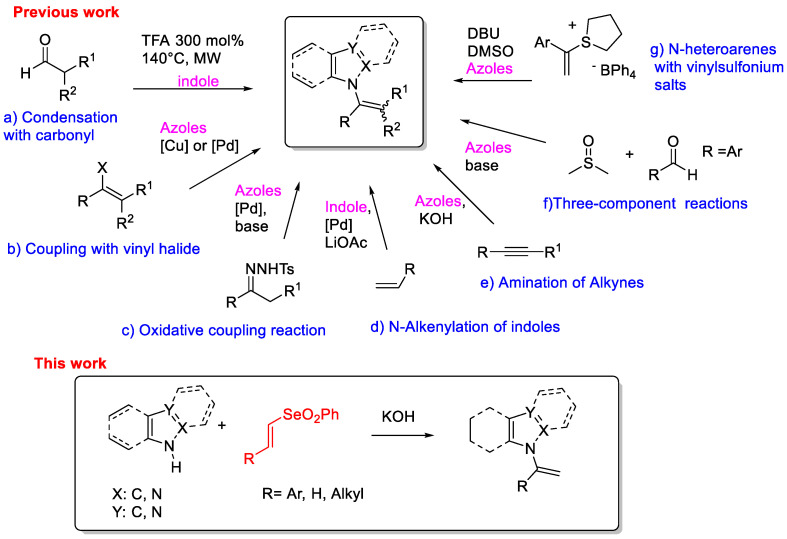
Synthetic approaches toward *N*-vinylazoles.

Owing to the widespread application of *N*-vinyl azoles, many strategies have been developed for their synthesis, and selected examples are reported in Scheme 1. A convenient route is the direct condensation of aldehydes on *N*-H indoles in the presence of a Brønsted or Lewis acid (Scheme 1, route a) [12]. This method requires harsh reaction conditions causing low functional group tolerance. With the vigorous growth of organometallic chemistry, the number of methods for the synthesis of *N*-vinyl azoles by metal-catalyzed cross-coupling reactions has increased over the years. A reliable method is copper- or palladium-catalyzed *N*-vinylation of azoles with vinyl bromide (Scheme 1, route b) [13,14,15]. *N*-vinyl indoles were also prepared through a palladium-catalyzed oxidative cross-coupling reaction of indoles with *N*-tosylhydrazones (Scheme 1, route c) [16,17] or by direct reaction with alkenes (Scheme 1, route d) [18]. All transition metal-catalyzed reactions have some limitations such as expensive catalysts/ligands and the preparation of the specific starting materials. Therefore, it is desirable to develop new types of coupling reactions that may circumvent these drawbacks. Base-mediated hydroamination of alkynes represents an alternative approach for the preparation of *N*-vinyl azoles [19,20,21] (Scheme 1, route e). Very recently, *N*-vinyl azoles were also obtained employing vinyl sulfonium salts in presence of a base [22,23] (Scheme 1, route f) or through a three-component reaction between aromatic aldehydes, dimethyl sulfoxide DMSO and azoles (Scheme 1, route g) [24].

On these grounds, and inspired by renewed interest in vinyl selenones due to their useful applications in challenging fields of organic synthesis [25,26,27,28], we decided to synthesize *N*-vinyl azoles employing these derivatives in a domino process. Although their chemistry shows analogies with that of the vinyl sulfones, their reactivity presents marked differences. Both contain an electron-withdrawing group that activates the double bond to the conjugate nucleophilic attack, but the weak C–Se bond donates to the phenylselenonyl moiety a better leaving group character for further substitution or elimination reactions. While several Michael addition/cyclization cascade reactions using vinyl selenones are widely reported in the literature [29,30,31], also in asymmetrical versions [32,33,34,35,36,37], only sporadic examples involving addition/elimination domino processes are investigated [38,39].

Herein we report a new application of the chemistry of vinyl selenones to an addition/elimination cascade process using mono-, bi- and tri-cyclic azoles with variously substituted vinyl selenones in presence of base.

## 2. Results and Discussion

The vinyl phenyl selenones necessary for the present investigation were synthesized starting from the corresponding vinyl selenides using Oxone^®^ as oxidant [40]. Initially, we explored the vinylation of indole **1a** with the phenyl vinyl selenone **2a** as a model reactant. As shown in Table 1, different inorganic and organic bases and different polar and apolar solvents were employed, and the best result was obtained using potassium hydroxide (2.5 equiv.) as base and *N*, *N*-dimethylformamide (DMF) as solvent (entry 5, 86% yield). Other dipolar aprotic solvents such as dimethyl sulfoxide or acetonitrile permit the progress of the reaction (entries 6–7), while the reaction did not occur in dichloromethane or in ethanol (entries 3–4). The substitution of potassium hydroxide with other inorganic bases such as cesium carbonate or sodium hydride gave the product **3a** in lower yields and longer reaction times (entries 8–9). Employing organic bases, the reaction proceeded with a yield of 79% with potassium *tert*-butoxide, while it did not proceed with 1,5-diazabiciclo[5.4.0]undec-7-ene DBU (entries 10–11). Conducting the reaction using a lower amount of potassium hydroxide (1.5 equiv.), the desirable product **3a** was isolated only in 40% yield (entry 12) and its formation was not observed in the absence of base (entry 13).

**Scheme 2:**
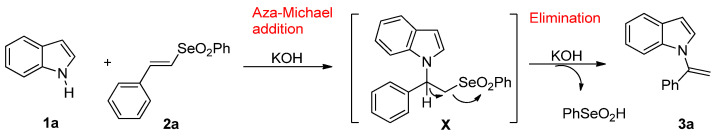
Plausible mechanism of Michael addition/elimination cascade.

The formation of the product **3a** can be explained through a *one-pot* reaction involving the addition/elimination process depicted in Scheme 2. An initial aza-Michael addition of indole **1a** to vinyl selenone **2a** forms the adduct X, which undergoes β-elimination of phenylseleninic acid to afford the product **3a**. The choice of base and solvent is crucial to the success of the reaction. As expected [41], the formation of more ionic salts, such as the potassium salt, and the use of highly coordinating solvents, such as the DMF, favor the N–alkylation of indole in the aza-Michael addition. Moreover, an excess of a strong base and the presence of an aprotic polar solvent is required to assist the subsequent E_2_ elimination step [22]. While the elimination of selenoxides is a well-known process in organochalcogen chemistry [42], the same reaction carried out on the selenones is much less common [43,44,45], hence the interest in exploring the result.

With the optimized reaction conditions, we evaluated the versatility of the methodology. Firstly, we tested the reactivity of the indole with aryl vinyl selenones. As highlighted in Table 2, various indoles bearing different functional groups such as CH_3_, Br, I, CH_2_OH, CN and CO_2_Et were investigated. Some of these substituents may provide the possibility of further synthetic transformations.

*N*-vinyl indoles **3a**–**b** were isolated in excellent yields using phenyl (*E*)-2-phenylvinyl selenone **2a**. Different aryl vinyl selenones **2b**–**e** bearing electron-deficient groups (R^2^ = 4-Cl–C_6_H_4_) and electron-rich groups (R^2^ = 4-CH_3_O–C_6_H_4_, 4-CH_3_–C_6_H_4_, 2-CH_3_–C_6_H_4_) have been successfully used, affording the corresponding *N*-vinyl indoles **3c**–**g** in good yields. A better yield was obtained starting from the selenone **2b** bearing an electron-deficient group in *para* position of the phenyl ring (**3c**, 87% yield), while when starting from the selenone **2e** bearing a methyl group in *ortho* position, the lowest yield (**3f**, 45%) was observed, probably due to steric hindrance. Interestingly, starting from (5-bromo-1*H*-indol-2yl) methanol, the exclusive formation of compound **3h** was achieved, demonstrating that when using a *bis* nucleophile there is also no trace of the Michael addition/cyclization cascade product. This result is reasonably a consequence of the high stability of the resulting conjugate system.

It is worth noting that when an unsubstituted vinyl selenone (**2f**, R^2^ = H) was used, the corresponding *N*-vinyl indoles **3i**–**l** were obtained in satisfactory yields, despite the formation of a less stable alkene. However, in this case the (5-bromo-1*H*-indol-2yl) methanol afforded the biologically relevant 8-bromo-3,4-dihydro-1*H*-[1,4]oxazino [4,3-*a*]indole (22% yield), as a result of a domino Michael/intramolecular nucleophilic substitution pathway [30], beside the expected *N*-vinyl indole **3l** (36% yield).

Encouraged by the results obtained with the indole scaffold, and in order to expand the substrate scope of the method, we proceeded to apply the same procedure to other azoles. As reported in Table 3, we first explored reactions with other benzo-fused mono-, di- and tri-azoles such as carbazole, benzoimidazole and benzotriazole and then with monocyclic azoles such as pyrrole, imidazole and pyrazole.

When the reaction was carried out on a carbazole nucleus employing aryl and unsubstituted vinyl selenones, the *N*-vinyl carbazoles **5a**–**b** were obtained in acceptable yields. *N*-vinyl benzimidazoles **5c**–**d** were obtained in good yields using benzoimidazole derivatives with aryl vinyl selenones. Employing benzotriazole with unsubstituted vinyl selenone, we observed the formation of *N*-vinyl benzotriazole **5e**, even if with a lower yield. The low reactivity of benzotriazole can be a consequence of its poor nucleophilicity as reported in the literature [13]. Switching to monocyclic azoles, *N*-vinyl pyrroles **5f**–**g**, *N*-vinyl imidazoles **5h**–**j** and *N*-vinyl pyrazoles **5k**–**l** were isolated in good yields using different aromatic and aliphatic vinyl selenones.

**Scheme 3:**
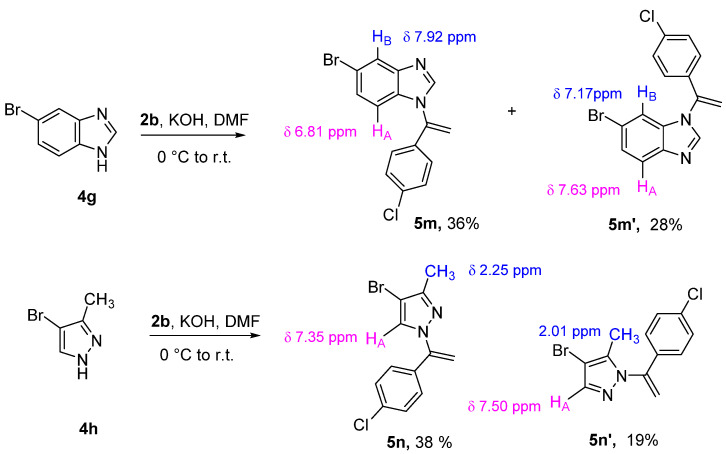
Vinylation of unsymmetrical N-diazoles.

As shown in Scheme 3, the *N*-vinylation reaction of unsymmetrical benzo-fused or monocyclic diazoles can lead to two regioisomers due to the functionalization at the NH position of the two tautomers. The *N*-vinyl benzoimidazole regioisomers **5m** and **5m′** were isolated when the 5-bromo benzoimidazole **4g** was employed as nucleophile. These compounds were separated in pure form by column chromatography in an almost 1:1 ratio. Structures were assigned according to the coupling constants and the shielding effects observed in their ^1^H NMR spectra. In both cases, the 4-chloro phenyl group causes a shielding effect on the benzoimidazole proton falling into its shielding cone. In particular, H_A_ appears as a doublet at 6.81 ppm (d, ^3^*J* = 8.6 Hz) in the compound **5m** and at 7.63 ppm (d, ^3^*J* = 8.6 Hz) in the compound **5m′**, while the proton H_B_ absorbs at 7.92 ppm (d, ^4^*J* = 1.1 Hz) in the compound **5m** and 7.17 ppm (d, ^4^*J* = 1.9 Hz) in the compound **5m’**. The structural assignment was confirmed by NOESY experiments (see supporting information). Similarly, the *N*-vinylation of the 4-bromo-3-methyl-1*H*-pyrazole **4h** led to the two regioisomers **5n** and **5n′** in a 2.5:1 ratio. The formation of **5n’** as minor isomer suggests that the steric hindrance of the methyl group plays a significant role in the N-functionalization.

## 3. Materials and Methods

### 3.1. General Information

Solvents and reagents were used as received unless otherwise noted. Thin-layer chromatography (TLC) was performed on silica gel 60 F254 (Merck, KGaA, Darmstadt, Germany). The products of the reactions were purified by normal chromatography column using Silica Gel Kiesegel 60 (70–230 mesh). Yields corresponded to isolated compounds. Melting points were determined in Kofler melting apparatus and values are uncorrected. All synthesized compounds were characterized by ^1^H NMR and ^13^C NMR spectroscopy. NMR experiments were obtained at 25 °C on a Bruker Avance at 400 MHz spectrometer, a Bruker Avance NEO 600 MHz spectrometer or a Bruker DPX 200 MHz spectrometer (Bruker, Billerica, MA, USA). Chemical shifts (δ) are reported in parts per million (ppm) in CDCl_3_ solution, if not otherwise specified. The following abbreviations are used to indicate multiplicity: s—singlet; d—doublet; t—triplet; q—quartet; quin—quintet; m—multiplet. Exact mass analyses were obtained by mass spectrometer Ion-Mobility QTof Agilent 6560 coupled with UHPLC 1290 Infinity II Agilent (UHPLC Agilent Technologies, Santa Clara, CA, USA).

### 3.2. Starting Materials

The indoles **1a**–**f** and other azoles **4a**–**h** used as starting products are commercially available, except for (5-bromo-1*H*-indol-2yl) methanol **1d** that was prepared following the method reported in the literature [30]. According to the literature procedures, starting vinyl selenones **2a**–**g** were prepared from the corresponding vinyl selenides by oxidation with an excess of Oxone [40].

### 3.3. General Procedure for the Synthesis of N-Vinyl Azoles

A stirred solution of N-indoles **1a**–**f** or other azoles **4a**–**h** (1 mmol) in DMF (2 mL) was treated with potassium hydroxide (2.5 equivalents) at 0 °C under argon atmosphere. After 10 min, a solution of the vinyl selenones **2a**–**g** (1 mmol) in DMF (2 mL) was added at 0 °C and the reaction mixtures were allowed to warm to room temperature. The progress of the reaction was monitored by TLC (petroleum ether/ethyl acetate 80:20), verifying the disappearance of the starting product. The reaction mixture was extracted with ethyl acetate (3 × 5 mL) and organic phase was then washed with H_2_O (3 × 5 mL). After drying with Na_2_SO_4_, the organic extracts were filtered and evaporated under reduced pressure. The products were purified using column chromatography on silica gel affording the N-vinyl indoles **3a**–**l** and other N-vinyl azoles **5a**–**n**, **5m′–5n′**.

*1-(1-Phenylvinyl)-1H-indole*, **3a** [24]: The crude product was purified by silica gel chromatography (petroleum ether: ethyl acetate 90:10 as eluent mixture) to afford **3a** as a white solid (m.p. 92–94 °C) in 82% yield. R.f. 0.80 (petroleum ether: ethyl acetate 80:20). ^1^H NMR (200 MHz, CDCl_3_, 25 °C, TMS): δ 7.71–7.66 (m, 1H), 7.42–7.32 (m, 5H), 7.21 (d, *J* = 3.3 Hz, 1H), 7.17–7.11 (m, 3H), 6.64 (d, *J* = 3.3 Hz, 1H), 5.61 (br s, 1H, C*H*H=), 5.40 (br s, 1H, CH*H*=).

*3-Methyl-1-(1-phenylvinyl)-1H-indole*, **3b** [24]: The crude product was purified by silica gel chromatography (petroleum ether: ethyl acetate 90:10 as eluent mixture) to afford **3b** as a yellow oil in 86% yield. R.f. 0.75 (petroleum ether: ethyl acetate 80:20). ^1^H NMR (400 MHz, CDCl_3,_ 25 °C, TMS): δ 7.62 (d, *J* = 7.3 Hz, 1H), 7.41–7.38 (m, 5H), 7.17–7.14 (m, 3H), 6.98 (s, 1H), 5.52 (br s, 1H, C*H*H=), 5.35 (br s, 1H, CH*H*=), 2.38 (s, 3H, CH_3_).

*1-[1-(4-Chlorophenyl)vinyl]-1H-indole*, **3c [16]**: The crude product was purified by silica gel chromatography (petroleum ether: ethyl acetate 98:2 as eluent mixture:) to afford **3c** as a white solid (m.p. 68–70 °C) in 87% yield. R.f. 0.83 (petroleum ether: ethyl acetate 80:20). ^1^H NMR (400 MHz, Chloroform-d): δ 7.61–7.54 (m, 1H), 7.28–7.21 (m, 2H), 7.20–7.14 (m, 2H), 7.08 (d, *J* = 3.3 Hz, 1H), 7.07–7.01 (m, 3H), 6.55 (dd, *J* = 3.3, 0.6 Hz, 1H), 5.50 (br s, 1H, C*H*H=), 5.31 (br s, 1H, CH*H*=).

*1-[1-(4-Methoxyphenyl)vinyl]-1H-indole*, **3d [24]**: The crude product was purified by silica gel chromatography (petroleum ether: ethyl acetate 90:10 as eluent mixture) to afford **3d** as a yellow solid (m.p. 51–53 °C) in 60% yield. R.f. 0.72 (petroleum ether: ethyl acetate 80:20). ^1^H NMR (200 MHz, CDCl_3,_ 25 °C, TMS): δ 7.64–7.58 (m, 1H), 7.24–7.04 (m, 6H), 6.86–6.79 (m, 2H), 6.57 (d, *J =* 3.25 Hz, 1H), 5.44 (br s, 1H, C*H*H=), 5.22 (br s, 1H, CH*H*=), 3.78 (s, 3H, CH_3_O).

*1-[1-(4-Methylphenyl)vinyl]-1H-indole*, **3e [17]**: The crude product was purified by silica gel chromatography (petroleum ether: ethyl acetate 97:3 as eluent mixture) to obtain **3e** as a dark brown solid (m.p. 52–55 °C) in 65% yield. R.f. 0.88 (petroleum ether: ethyl acetate 80:20). ^1^H NMR (400 MHz, Chloroform-d): δ 7.61–7.53 (m, 1H), 7.16–6.98 (m, 8H), 6.53 (d, *J* = 3.4 Hz, 1H), 5.46 (br s, 1H, C*H*H=), 5.23 (br s, 1H, CH*H*=), 2.29 (s, 3H, CH_3_).

*1-[1-(2-Methylphenyl)vinyl]-1H-indole*, 3f [24]: The crude was purified by silica gel chromatography (petroleum ether: ethyl acetate 97:3 as eluent mixture) to obtain **3f** as a yellow oil in 45% yield. R.f. 0.86 (petroleum ether: ethyl acetate 80:20). ^1^H NMR (400 MHz, Chloroform-d): δ 7.58–7.50 (m, 1H), 7.36 (d, *J* = 8.7 Hz, 1H), 7.29–7.19 (m, 3H), 7.10–7.01 (m, 3H), 6.97–6.93 (m, 1H), 6.50–6.46 (m, 1H), 5.42 (br s, 1H, C*H*H=), 5.12 (br s, 1H, CH*H*=), 1.76 (s, 3H, CH_3_).

*1-[1-(2-Methylphenyl)vinyl]-1H-indole-4-carbonitrile*, **3g**: The crude was purified by silica gel chromatography (petroleum ether: ethyl acetate 90:10 as eluent mixture) to obtain **3g** as a greenish oil in 71% yield. R.f. 0.60 (petroleum ether: ethyl acetate 80:20). ^1^H NMR (400 MHz, Chloroform-*d*): δ 7.42–7.15 (m, 6H), 7.12–7.02 (m, 2H), 6.72 (d, *J* = 3.4, 0.9 Hz, 1H), 5.44 (br s, 1H, C*H*H=), 5.23 (br s, 1H, CH*H*=), 1.73 (s, 3H, CH_3_). ^13^C NMR (100 MHz, CDCl_3_): δ 144.7, 136.7, 136.5, 135.3, 130.9, 130.1, 130.3, 130.2, 129.7, 126.5, 125.8, 122.1, 118.6, 116.4, 108.6, 103.7, 102.5, 19.3. HRMS (ESI Q-TOF): *m/z* [M+H]^+^ calculated for C_18_H_15_N_2_ 259.1230; found 259.1225.

*{5-Bromo-1-[1-(4-chlorophenyl)vinyl]-1H-indol-2-yl}methanol*, **3h**: The crude was purified by silica gel chromatography (petroleum ether: ethyl acetate 75:25 as eluent mixture) to obtain **3h** as a yellow oil in 50% yield. R.f. 0.25 (petroleum ether: ethyl acetate 80:20). ^1^H NMR (400 MHz, Chloroform-*d*): δ 7.68 (d, *J* = 1.9 Hz, 1H), 7.23–7.16 (m, 2H), 7.13 (dd, *J* = 8.7, 1.9 Hz, 1H), 7.01–6.91 (m, 2H), 6.89 (d, *J* = 8.7 Hz, 1H), 6.52 (s, 1H), 5.97 (br s, 1H, C*H*H=), 5.45 (br s, 1H, CH*H*=), 4.53 (br s, 2H, CH_2_). ^13^C NMR (100 MHz, CDCl_3_): δ 141.6, 140.8, 137.0, 135.5, 135.0, 129.4, 129.3 (2C), 127.1 (2C), 125.6, 123.4, 114.6, 113.8, 112.5, 102.5, 57.5. HRMS (ESI Q-TOF): *m/z* [M+H]^+^ calculated for C_17_H_14_BrClNO 361.9942; found 361.9924.

*1-Vinyl-1H-indole*, **3i** [9]: The crude was purified by silica gel chromatography (petroleum ether: ethyl acetate 97:3 as eluent mixture) to obtain **3i** as whitish crystalline solid (m. p. 34–36 °C) in 60% yield. R.f. 0.75 (petroleum ether: ethyl acetate 80:20). ^1^H NMR (600MHz, Chloroform-*d*): δ 7.54 (d, *J* = 7.9 Hz, 1H), 7.40 (dd, *J* = 8.3, 1.0 Hz, 1H), 7.36 (d, *J* = 3.4 Hz, 1H), 7.19–7.13 (m, 2H, *H*ar, C*H*=), 7.10–7.06 (m, 1H), 6.56 (d, *J* = 3.4 Hz, 1H), 5.12 (dd, *J* = 15.7, 1.4 Hz, 1H, C*H*H=), 4.70 (dd, *J* = 8.9, 1.4 Hz, 1H, CH*H*=).

*Ethyl 5-bromo-1-vinyl-1H-indole-2-carboxylate*, **3j**: The crude was purified by silica gel chromatography (petroleum ether: ethyl acetate 97:3 as eluent mixture) to obtain **3j** as a yellowish oil in 35% yield. R.f. 0.77 (petroleum ether: ethyl acetate 80:20). ^1^H NMR (400 MHz, Chloroform-*d*): δ 7.47 (d, *J* = 1.9 Hz, 1H), 7.69–7.59 (m, 2H, Har, C*H*=), 7.45 (dd, *J* = 9.0, 1.9 Hz, 1H), 7.03 (s, 1H), 5.40 (dd, *J* = 15.8, 1.2 Hz, 1H, C*H*H=), 5.31 (dd, *J* = 8.7, 1.2 Hz, 1H, CH*H*=), 7.01 (quart, *J* = 7.1Hz, 2H, CH_2_O), 1.38 (t, *J* = 7.1 Hz, 3H, CH_3_). ^13^C NMR (100 MHz, CDCl_3_): δ 161.5, 136.7, 132.1, 129.0, 128.7, 128.6, 125.1, 114.7, 114.1, 111.1, 108.2, 60.7, 13.2. HRMS (ESI Q-TOF): *m/z* [M+H]^+^ calculated for C_13_H_13_BrNO_2_ 294.0124; found 294.0114.

*5-Iodo-1-vinyl-1H-indole*, **3k**: The crude was purified by silica gel chromatography (petroleum ether: ethyl acetate 90:10 as eluent mixture) to obtain **3k** as a white powder (m.p. 62–67 °C) in 50% yield. R.f. 0.80 (petroleum ether: ethyl acetate 80:20). ^1^H NMR (400 MHz, Chloroform-*d*): δ 7.87 (d, *J* = 1.7 Hz, 1H), 7.43 (dd, *J* = 8.6, 1.7 Hz, 1H), 7.30 (d, *J* = 3.4 Hz, 1H), 7.20–7.13 (m, 1H), 7.07 (dd, *J* = 15.7, 8.9 Hz, 1H, C*H*=), 6.47 (d, *J* = 3.4 Hz, 1H), 5.13 (dd, *J* = 15.7, 1.5 Hz, 1H, C*H*H=), 4.74 (dd, *J* = 9.0, 1.5 Hz, 1H, CH*H*=). ^13^C NMR (100 MHz, CDCl_3_): δ 134.7, 131.6, 131.1, 130.1, 129.4, 124.3, 111.6, 104.2, 97.7, 84.3. HRMS (ESI Q-TOF): *m/z* [M+H]^+^ calculated for C_10_H_9_IN 269.9774; found 269.9772.

*(5-Bromo-1-vinyl-1H-indol-2-yl)methanol*, **3l**: The crude was purified by silica gel chromatography (petroleum ether: ethyl acetate 70:30 as eluent mixture) to obtain **3l** as a yellow solid (m.p.88–93 °C) in 36% yield. R.f. 0.23 (petroleum ether: ethyl acetate 80:20). ^1^H NMR (400 MHz, Chloroform-*d*): δ 7.63 (d, *J* = 2.0 Hz, 1H), 7.41 (d, *J* = 8.7 Hz, 1H), 7.25 (dd, *J* = 8.8, 2.0 Hz, 1H), 7.11 (dd, *J* = 15.9, 9.1 Hz, 1H, C*H*=), 6.41 (s, 1H), 5.43 (dd, *J* = 15.9, 1.0 Hz, 1H, C*H*H=), 5.09 (dd, *J* = 9.1, 0.9 Hz, 1H, CH*H*=), 4.74 (d, *J* = 4.0 Hz, 2H, CH_2_O). ^13^C NMR (100 MHz, CDCl_3_): δ 139.4, 135.5, 130.1, 129.9, 126.0, 123.6, 114.1, 112.9, 105.4, 103.7, 57.6 HRMS (ESI Q-TOF): *m/z* [M]^+^ calculated for C_11_H_10_BrNO 250.9940; found 250.9942.

*8-Bromo-3,4-dihydro-1H-*[1,4]*oxazino [4,3-a]indole* [30]: The crude was purified by silica gel chromatography (petroleum ether: ethyl acetate 80:20 as eluent mixture) to obtain a white solid (m.p. 158–159 °C) in 22% yield. R.f. 0,46 (petroleum ether: ethyl acetate 80:20). ^1^H NMR (400 MHz, Chloroform-*d*): δ 7.61 (d, *J* = 1.9 Hz, 1H), 7.20–7.16 (m, 1H), 7.07 (d, *J* = 8.6 Hz, 1H), 6.08 (s, 1H), 4.90 (s, 2H), 4.11–3.96 (m, 4H).

*9-[1-(2-Methylphenyl)vinyl]-9H-carbazole*, **5a**: The crude was purified by silica gel chromatography (petroleum ether:ethyl acetate 90:10 as eluent mixture) to obtain **5a** as a white solid (m.p. 76–80 °C) in 40% yield. R.f. 0.93 (petroleum ether: ethyl acetate 80:20). ^1^H NMR (400 MHz, Chloroform-*d*): δ 8.01 (dt, *J* = 7.7, 1.0 Hz, 2H), 7.43 (dd, *J* = 6.9, 2.3 Hz, 1H), 7.28–7.12 (m, 8H), 7.00 (dd, *J* = 6.4, 2.4 Hz, 1H), 5.63 (br s, 1H, C*H*H=), 5.60 (br s, 1H, CH*H*=), 1.68 (s, 3H, CH_3_). ^13^C NMR (100 MHz, CDCl_3_): δ 143.6, 140.3 (2C), 137.5, 136.6, 131.1, 129.9, 129.1, 126.4, 126.0 (2C), 123.8 (2C), 120.2 (2C), 120.1 (2C), 113.3, 111.3 (2C), 20.1. HRMS (ESI Q-TOF): *m/z* [M+H]^+^ calculated for C_21_H_18_N 284.1434; found 284.1434.

*9-Vinyl-9H-carbazole*, **5b** [19]: The crude was purified by silica gel chromatography (petroleum ether:ethyl acetate 90:10 as eluent mixture) to obtain **5b** as a white solid (m.p. 64–65 °C) in 50% yield. R.f. 0.74 (petroleum ether: ethyl acetate 80:20). ^1^H NMR (400 MHz, Chloroform-*d*): δ 8.00 (dt, *J* = 7.7, 1.0 Hz, 2H), 7.59 (dt, *J* = 8.3, 0.9 Hz, 2H), 7.40 (ddd, *J* = 8.3, 7.2, 1.3 Hz, 2H), 7.29–7.17 (m, 3H, 2*H*ar, C*H*=), 5.48 (dd, *J* = 15.9, 0.9 Hz, 1H, C*H*H=), 5.09 (dd, *J* = 9.2, 0.9 Hz, 1H, CH*H*=).

*1-(1-Phenylvinyl)-1H-benzimidazolo*, **5c** [24]: The crude was purified by silica gel chromatography (petroleum ether: ethyl acetate 50:50 as eluent mixture) to obtain **5c** as a yellow solid (m.p. 57–58 °C) in 77% yield. R.f. 0.58 (petroleum ether: ethyl acetate 40:60). ^1^H NMR (400 MHz, CDCl_3_, 25 °C, TMS): δ 8.01 (s, 1H), 7.89 (d, *J* = 8.1 Hz, 1H), 7.45–7.21 (m, 7H), 7.10 (d, *J* = 8.1 Hz, 1H), 5.72 (s, 1H), 5.50 (s, 1H).

*1-[1-(4-Chlorophenyl)vinyl]-1H-1,3-benzimidazole*, **5d**: The crude was purified by silica gel chromatography (petroleum ether: ethyl acetate 50:50 as eluent mixture) to obtain **5d** as a white solid (m.p. 78–81 °C) in 66% yield. R.f. 0.38 (petroleum ether: ethyl acetate 70:30), ^1^H NMR (400 MHz, Chloroform-*d*): δ 7.94 (s, 1H), 7.78 (d, *J* = 8.0 Hz, 1H), 7.31–7.09 (m, 6H), 6.96 (d, *J* = 8.1 Hz, 1H), 5.61 (d, *J* = 1.1 Hz, 1H, C*H*H=), 5.41 (d, *J* = 1.1 Hz, 1H, CH*H*=). ^13^C NMR (100 MHz, CDCl_3_): δ 144.1, 143.1, 141.4, 136.0, 133.9, 133.7, 129.3 (2C), 128.2 (2C), 123.7, 123.0, 120.7, 111.8, 110.1 HRMS (ESI Q-TOF): *m/z* [M+H]^+^ calculated for C_15_H_12_ClN_2_ 255.0684; found 255.0679.

*1-Vinyl-1H-1,2,3-benzotriazole*, **5e**: The crude was purified by silica gel chromatography (petroleum ether: ethyl acetate 95:5 as eluent mixture) to obtain **5e** as a yellowish oil in 30% yield. R.f. 0.48 (petroleum ether: ethyl acetate 80:20). ^1^H NMR (400 MHz, Chloroform-*d*): δ 8.04 (d, *J* = 8.2 Hz, 1H), 7.64 (d, *J* = 8.4 Hz, 1H), 7.57–7.47 (m, 2H, Har, C*H*=), 7.36 (ddd, *J* = 8.1, 7.0, 1.0 Hz, 1H), 5.90 (dd, *J* = 16.0, 1.5 Hz, 1H, C*H*H=), 5.21 (dd, *J* = 9.2, 1.6 Hz, 1H, CH*H*=). ^13^C NMR (100 MHz, CDCl_3_): δ 146.5, 131.6, 129.5, 128.5, 124.7, 120.6, 110.3, 104.2. HRMS (ESI Q-TOF): *m/z* [M+H]^+^ calculated for C_8_H_8_N_3_ 146.0713; found 145.0713.

*1-(1-Phenylvinyl)-1H-pyrrole*, **5f** [14]: The crude was purified by silica gel chromatography (petroleum ether: ethyl acetate 90:10 as eluent mixture) to obtain **5f** as a yellowish oil in 53% yield. R.f. 0.78 (petroleum ether: ethyl acetate 80:20). ^1^H NMR (400 MHz, CDCl_3_, 25 °C, TMS): δ 7.43–7.41 (m, 5H), 6.84 (t, *J* = 2.15 Hz, 2H), 6.28 (t, *J* = 2.15 Hz, 2H), 5.20 (br s, 1H, C*H*H=), 5.12 (br s, 1H, CH*H*=).

*1-[1-(2-Methylphenyl)vinyl]-1H-pyrrole*, **5g**: The crude was purified by silica gel chromatography (petroleum ether:ethyl acetate 90:10 as eluent mixture) to obtain **5g** as a colorless oil in 53% yield. R.f. 0.81 (petroleum ether: ethyl acetate 80:20). ^1^H NMR (400 MHz, Chloroform-*d*): δ 7.24 (td, *J* = 8.8, 1.7 Hz, 2H), 7.18–7.09 (m, 2H), 6.63 (t, *J* = 2.2 Hz, 2H), 6.13 (t, *J* = 2.3 Hz, 2H), 5.23 (br s, 1H, C*H*H=), 4.66 (br s, 1H, CH*H*=), 1.97 (s, 3H, CH_3_). ^13^C NMR (100 MHz, CDCl_3_): δ 145.8, 137.3, 136.9, 130.4, 130.3, 129.1, 125.9, 119.6 (2C), 109.9 (2C), 101.2, 19.1. HRMS (ESI Q-TOF): *m/z* [M+H]^+^ calculated for C_13_H_14_N 184.1121; found 184.1116.

*1-(1-Phenylvinyl)-1H-imidazole*, **5h** [14]: The crude was purified by silica gel chromatography (petroleum ether: ethyl acetate 20:80 as eluent mixture) to obtain **5h** as a yellow oil in 63% yield. R.f. 0.58 (petroleum ether: ethyl acetate 20:80). ^1^H NMR (400 MHz, CDCl_3,_ 25 °C, TMS): δ 7.74 (s, 1H), 7.45–7.38 (m, 5H), 7.16–7.13 (m, 1H), 7.06–7.03 (m, 1H), 5.35 (br s, 1H, C*H*H=), 5.32 (br s, 1H, CH*H*=).

*1-[1-(2-Methylphenyl)vinyl]-1H-imidazole*, **5i**: The crude was purified by silica gel chromatography (dichloromethane: methanol 95:5 as eluent mixture) to obtain **5i** as a colorless oil with a 75% yield. R.f. 0.36 (dichloromethane: methanol 98:2). ^1^H NMR (400 MHz, Chloroform-*d*): δ 7.39 (s, 1H), 7.31–7.22 (m, 2H), 7.22–7.12 (m, 2H), 7.02 (br s, 1Har), 6.93 (br s, 1Har), 5.37 (br s, 1H, C*H*H=), 4.87 (br s, 1H, CH*H*=), 1.98 (s, 3H, CH_3_). ^13^C NMR (100 MHz, CDCl_3_): δ 142.9, 136.9, 136.2, 135.4, 130.8, 130.3, 130.2, 129.7, 126.3, 117.6, 104.7, 19.2. HRMS (ESI Q-TOF): *m/z* [M+H]^+^ calculated for C_12_H_13_N_2_ 185.1073; found 185.1071.

*1-(oct-1-en-2-yl)-1H-imidazolo*, **5j**: The crude was purified by silica gel chromatography (petroleum ether: ethyl acetate 20:80 as eluent mixture) to obtain **5j** as a yellow oil in 61% yield. R.f. 0.54 (petroleum ether: ethyl acetate 20:80). ^1^H NMR (400 MHz, CDCl_3,_ 25 °C, TMS): δ 7.71 (s, 1H), 7.10–7.15 (m, 2H), 5.08 (br s, 1H, C*H*H=), 4.82 (br s, 1H, CH*H*=), 2.51 (t, *J* = 7.1 Hz, 2H, CH_2_), 1.51 (quint, *J* = 7.1 Hz, 2H, CH_2_), 1.39–1.23 (m, 6H, 3CH_2_), 0.9 (t, *J* = 6.8 Hz, 3H, CH_3_). ^13^C NMR (100 MHz, CDCl_3,_ 25 °C, TMS): δ 142.9, 129.6 (2C), 116.9, 102.7, 33.8, 31.4, 28.5, 26.8, 22.4, 13.9. HRMS (ESI Q-TOF): *m/z* [M+H^+^] calculated for C_11_H_19_N_2_ 179.1543; found 179.1550.

*1-(1-(4-Chlorophenyl)vinyl)-1H-pyrazole*, **5k** [46]: The crude was purified by silica gel chromatography (petroleum ether: ethyl acetate from 90:10 to 80:20 as eluent mixture) to obtain **5k** as a yellow oil in 74% yield. R.f. 0,66 (petroleum ether: ethyl acetate from 80:20). ^1^H NMR (200 MHz, CDCl_3,_ 25 °C, TMS): δ 7.72 (s, 1H), 7.54 (d, *J* = 2.15 Hz, 1H), 7.45–7.29 (m, 4H), 6.41–6.39 (m, 1H), 5.59 (br s, 1H, C*H*H=), 5.23 (br s, 1H, CH*H*=).

*1-[1-(2-Methylphenyl)vinyl]-1H-pyrazole*, **5l**: The crude was purified by silica gel chromatography (petroleum ether: ethyl acetate 90:10 as eluent mixture) to obtain **5l** as a brownish oil in 73% yield. R.f. 0.75 (petroleum ether: ethyl acetate from 80:20). ^1^H NMR (400 MHz, Chloroform-*d*): δ 7.59 (d, *J* = 1.7 Hz, 1H), 7.27 (t, *J* = 7.4 Hz, 2H), 7.22–7.13 (m, 2H), 7.11 (dd, *J* = 2.5, 0.7 Hz, 1H), 6.24–6.19 (m, 1H), 5.79 (br s, 1H, C*H*H=), 4.82 (br s, 1H, CH*H*=), 1.99 (s, 3H, CH_3_). ^13^C NMR (100 MHz, CDCl_3_): δ 144.9, 141.2, 137.3, 135.7, 130.5, 130.5, 129.5, 128.7, 126.1, 106.9, 103.5, 19.2. HRMS (ESI Q-TOF): *m/z* [M+H^+^] calculated for C_12_H_13_N_2_ 185.1073; found 185.1077.

*5-Bromo-1-[1-(4-chlorophenyl)vinyl]-1H-1,3-benzimidazole*, **5m**: The crude was purified by silica gel chromatography (petroleum ether: ethyl acetate 60:40 as eluent mixture) to obtain **5m** as a brownish oil in 30% yield. R.f. 0.33 (petroleum ether: ethyl acetate 70:30). ^1^H NMR (400 MHz, Chloroform-*d*): δ 7.93 (s, 1H_C_), 7.92 (d, *J*= 1.1 Hz, 1H_B_), 7.32–7.26 (m, 2Har, AA’BB’ system), 7.24 (dd, *J* = 8.6, 1.8 Hz, 1H_D_), 7.18–7.11 (m, 2Har, AA’BB’ system), 6.81 (d, *J* = 8.6 Hz, 1H_A_), 5.63 (d, *J* = 1.2 Hz, 1H_E_, C*H*H=), 5.41 (d, *J* = 1.2 Hz, 1H_F_, CH*H*=). ^13^C NMR (100 MHz, CDCl_3_): δ 145.4 (C), 144.0 (CH), 141.1 (C), 136.3 (C), 133.5 (C), 132.7 (C), 129.4 (2CH), 128.1 (2CH), 126.9 (CH), 123.6 (CH), 116.1 (C), 113.0 (CH), 110.5 (CH_2_=). HRMS (ESI Q-TOF): *m/z* [M+H^+^] calculated for C_15_H_11_BrClN_2_ 332.9789; found 332.9803.

*6-Bromo-1-[1-(4-chlorophenyl)vinyl]-1H-1,3-benzimidazole*, **5m′**: The crude was purified by silica gel chromatography (petroleum ether: ethyl acetate 60:40 as eluent mixture) to obtain **5m’** as a yellowish oil in 30% yield. R.f. 0.28 (petroleum ether: ethyl acetate 70:30). ^1^H NMR (400 MHz, Chloroform-*d*): δ 7.89 (s, 1H_C_), 7.63 (d, *J* = 8.6 Hz, 1H_A_), 7.34 (dd, *J* = 8.6, 1.9 Hz, 1H_D_), 7.32–7.28 (m, 2Har, AA′BB′ system), 7.17 (d, *J* = 1.4Hz, 1H_B_), 7.15- 7.12 (m, 2Har, AA’BB’ system), 5.66 (d, *J* = 1.2 Hz, 1H_E_, C*H*H_=_), 5.42 (d, *J* = 1.2 Hz, 1H_F_, CH*H*=). ^13^C NMR (100 MHz, CDCl_3_): δ 143.6 (CH), 143.0 (C), 141.8 (C), 140.9 (C), 136.3 (C), 133.5 (C), 129.5 (2CH), 128.0 (2CH), 126.5 (CH), 122.0 (CH), 117.2 (C), 114.6 (CH), 110.9 (CH_2_=). HRMS (ESI Q-TOF): *m/z* [M+H^+^] calculated for C_15_H_11_BrClN_2_ 332.9789; found 332.9810.

*4-Bromo-1-[1-(4-chlorophenyl)vinyl]-3-methyl-1H-pyrazole*, **5n**: The crude was purified by silica gel chromatography (petroleum ether:ethyl acetate from 85:15 to 80:20 as eluent mixture) to obtain **5n** as a whitish oil with a 30% yield. R.f. 0.88 (petroleum ether: ethyl acetate 70:30). ^1^H NMR (400 MHz, chloroform-d): δ 7.35 (s,1H_A_), 7.32–7.28 (m, 2Har, AA’BB’ system), 7.24–7.19 (m, 2Har, AA’BB’ system), 5.45 (br s, 1H_F_, C*H*H=), 5.07 (br s, 1H_E_, CH*H*=, 2.25 (s, 3H, CH_3_). ^13^C NMR (100 MHz, CDCl_3_): δ 149.6 (C), 144.6 (C), 135.7 (C), 133.9 (C), 130.1 (CH), 129.5 (2CH), 129.0 (2CH), 105.2 (C), 96.1 (CH_2_=), 12.3 (CH_3_). HRMS (ESI Q-TOF): *m/z* [M+H^+^] calculated for C_12_H_11_BrClN_2_ 296.9789; found 296.9776.

*4-Bromo-1-[1-(4-chlorophenyl)vinyl]-5-methyl-1H-pyrazole*, **5n′**: The crude was purified by silica gel chromatography (petroleum ether: ethyl acetate 90:10 as eluent mixture) to obtain **5n’** as a yellowish oil with a 30% yield. R.f. 0.81 (petroleum ether: ethyl acetate 70:30). ^1^H NMR (400 MHz, Chloroform-*d*): δ 7.50 (s, 1H, H_A_), 7.28–7.22 (m, 2Har, AA’BB’ system), 7.07–7.01 (m, 2Har, AA′BB′ system), 5.70 (d, *J* = 1.0 Hz, 1H_E_, C*H*H=), 5.35 (d, *J* = 1.0 Hz, 1H_F_, CH*H*=), 2.01 (s, 3H, CH_3_). ^13^C NMR (100 MHz, CDCl_3_): δ 144.5 (C), 140.3 (CH), 138.3 (C), 135.5 (C), 134.4 (C), 129.1 (2CH), 127.6 (2CH), 113.0 (CH_2_=), 95.0 (C), 10.9 (CH_3_). HRMS (ESI Q-TOF): *m/z* [M+H^+^] calculated for C_12_H_11_BrClN_2_ 296.9789; found 296.9776.

## 4. Conclusions

In summary, we developed a novel method for the synthesis of N-vinyl azoles through a domino process. The selenonyl group plays a dual role by promoting the Michael addition and then acting as a leaving group in the one-pot elimination. This simple and metal-free approach employs easily accessible starting materials such as commercially available azoles, potassium hydroxide and bench-stable vinyl selenones. This protocol confirms the synthetic versatility of the vinyl selenones, opens the way to further studies concerning addition/elimination cascades and represents a simple and general way to synthesize a variety of particularly attractive N-vinyl heterocycles, making it a valuable addition to existing methods for their synthesis.

## Figures and Tables

**Table 1:** Optimization of the reaction conditions.

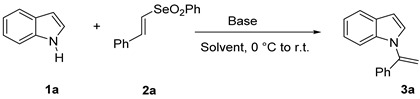						
Entry	Base	Equivalent	Solvent	Temp.	Time	3a, Yields %
1	Cs_2_CO_3_	2.5	Toluene	reflux	12 h	-
2	Li_2_CO_3_	2.5	CH_2_Cl_2_	r.t.	48 h	-
3	KOH	2.5	CH_2_Cl_2_	0 °C to r.t.	9 h	-
4	KOH	2.5	EtOH	0 °C to r.t.	60 h	-
5	KOH	2.5	DMF	0 °C to r.t.	2 h	86
6	KOH	2.5	DMSO	r.t.	12 h	82
7	KOH	2.5	CH_3_CN	0 °C to r.t.	12 h	50
8	Cs_2_CO_3_	2.5	DMF	0 °C to r.t.	72 h	51
9	NaH 60%	2.5	DMF	0 °C to r.t.	48 h	49
10	DBU	2.5	DMF	0 °C to r.t.	12 h	-
11	*t*-BuOK	2.5	DMF	0 °C to r.t.	12 h	79
12	KOH	1.5	DMF	0 °C to r.t.	72 h	40
13	-	-	DMF	0 °C to r.t.	12 h	-

DMSO: dimethyl sulfoxide; DMF: dimethyl formamide.

**Table 2:** Synthesis of *N*-vinyl indoles with different vinyl selenones.

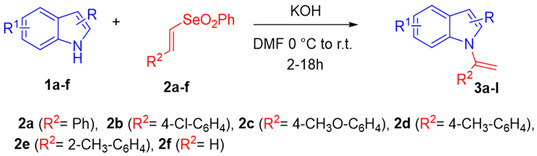			
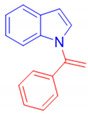	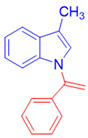	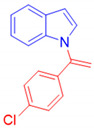	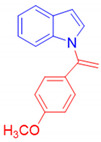
**3a**, 82%	**3b**, 86%	**3c**, 87%	**3d**, 60%
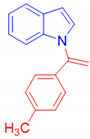	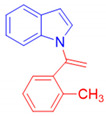	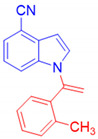	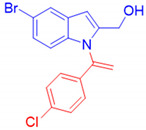
**3e**, 65%	**3f**, 45%	**3g**, 71%	**3h**, 50%
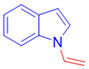	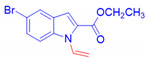	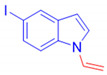	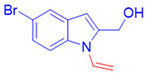
**3i**, 60%	**3j**, 35%	**3k**, 50%	**3l**, 36% Oxazine *, 22%

* 8-bromo-3,4-dihydro-1*H*-[1,4]oxazino[4,3-*a*]indole (22% yield).

**Table 3:** Vinylation of other *N*-heteroarenes substrates.

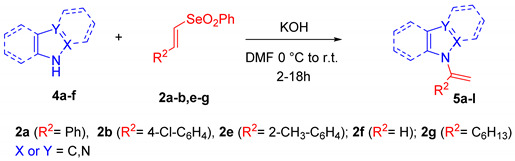			
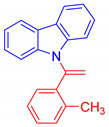	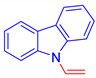	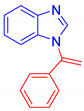	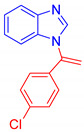
**5a**, 40%	**5b**, 50%	**5c**, 77%	**5d**, 66%
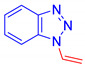	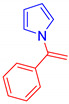	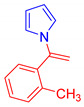	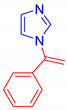
**5e**, 30%	**5f**, 53%	**5g**, 58%	**5h**, 63%
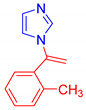	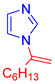	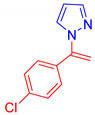	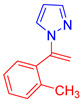
**5i**, 75%	**5j**, 61%	**5k**, 74%	**5l**, 73%

## Data Availability

The data presented in this study are available in the article and in Supplementary Materials.

## Supplementary Materials

The following supporting information can be downloaded at: https://www.mdpi.com/article/10.3390/molecules28166026/s1, Figures S1–S45: Copies of ^1^H and ^13^C NMR Spectra of Compounds **3a**–**l**, **5a**–**n**, **5m′**–**n′ [9,14,16,17,19,24,30,46]**; Figures S46–S49: NOESY experiments of compounds **5m**–**5m′** and **5n**–**5n′**.
