# Supplementary material for: Vinylation of N-Heteroarenes through Addition/Elimination Reactions of Vinyl Selenones
Source: Molecules. 2023 Aug 12;28(16):6026. doi: 10.3390/molecules28166026 (PMC10459632; doi:10.3390/molecules28166026)
Supplement: Supplementary file 1 [file molecules-28-06026-s001.zip › molecules-2470286-supplementary.pdf]

^{1}H and ^{13}C NMR spectra of compounds **3a-l** and **5a-n, 5m', 5n'** S2

NOESY correlation of compounds **5m, 5m', 5n, 5n'** S25

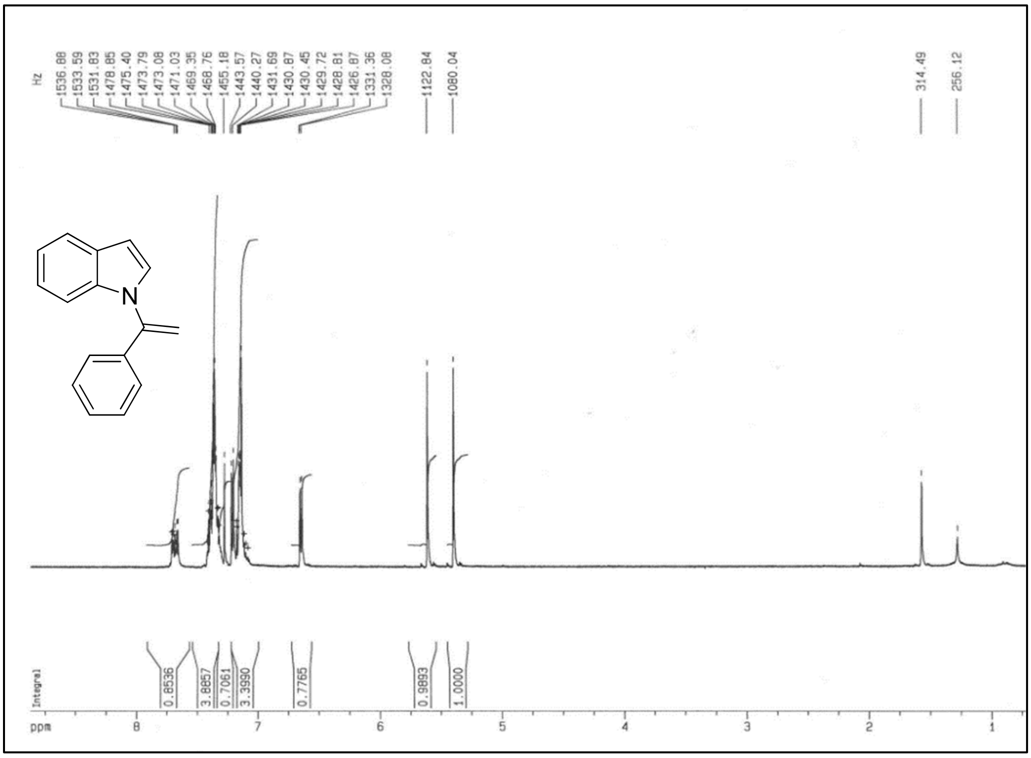

Figure S1. ^{1}H spectrum of compound **3a**[24] in $CDCl_3$.

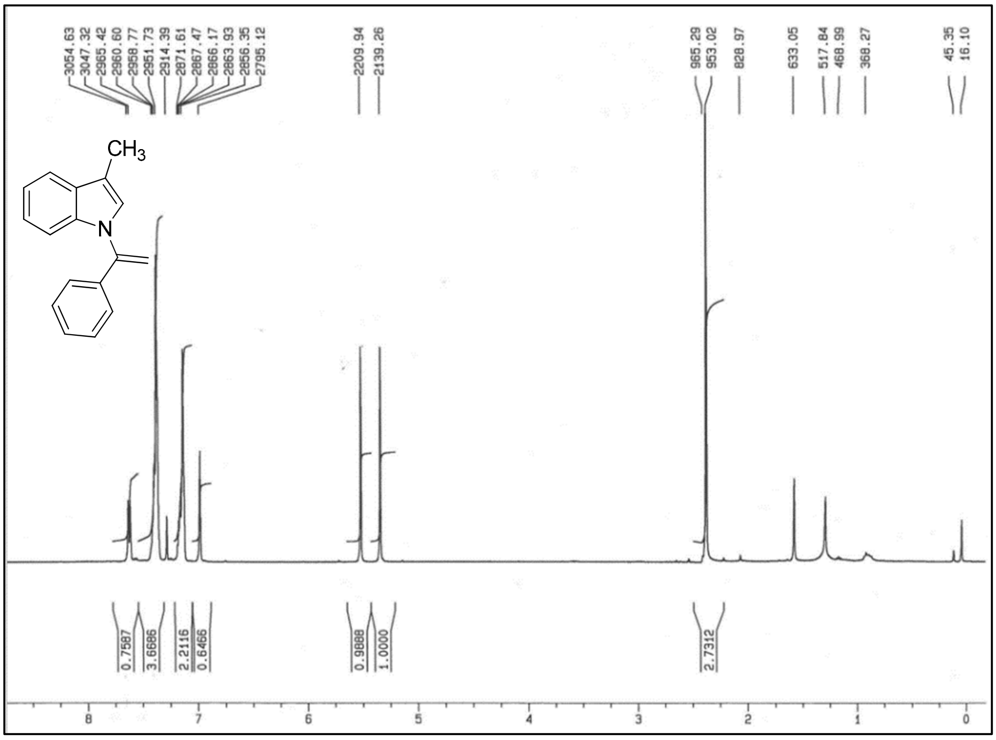

Figure S2. ^{1}H spectrum of compound **3b**[24] in $CDCl_3$.

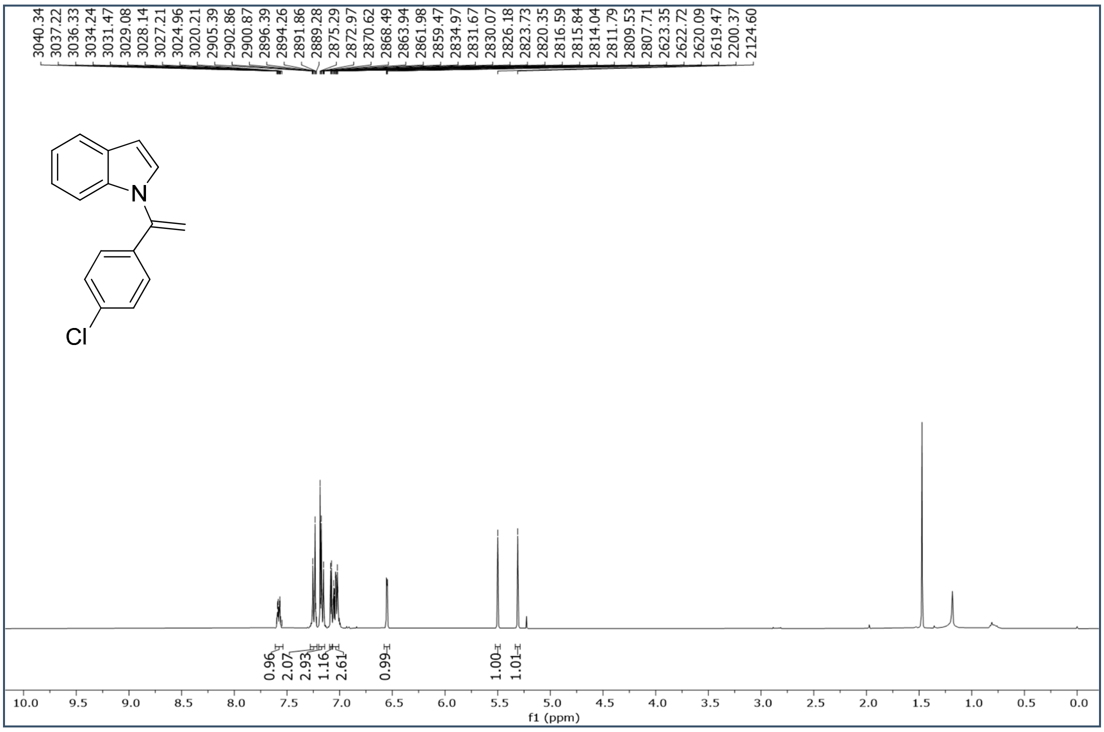

Figure S3. ^{1}H spectrum of compound **3c**[16] in $CDCl_3$.

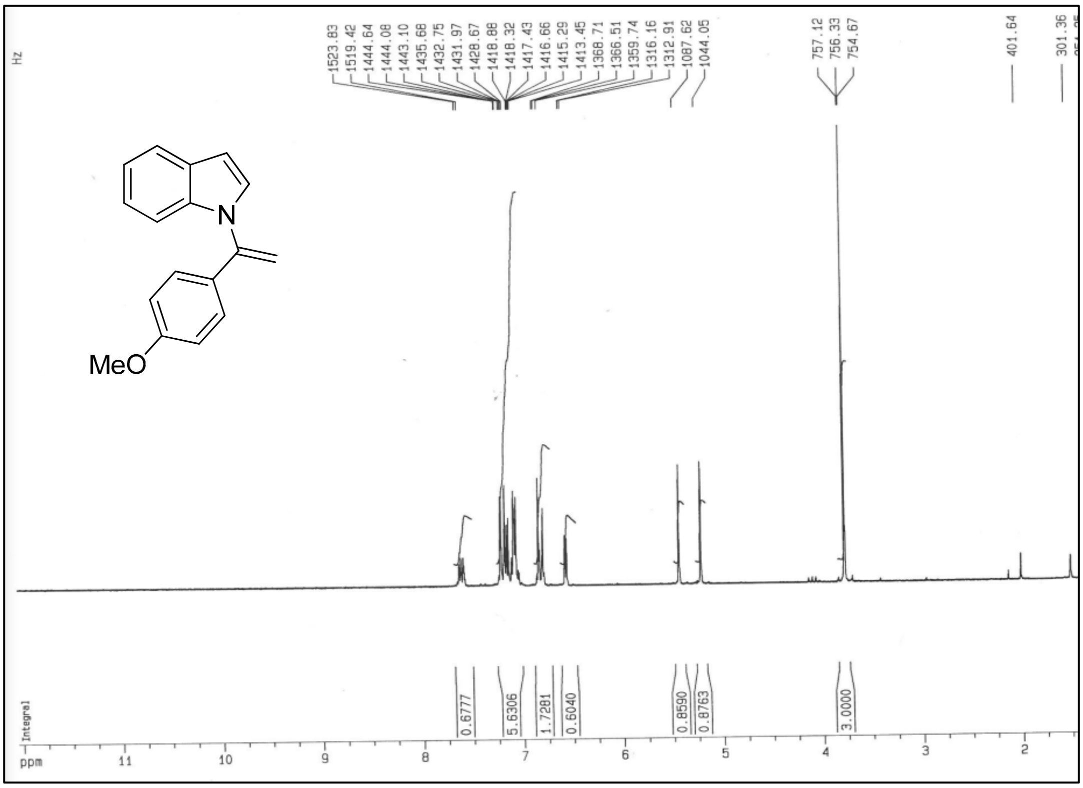

Figure S4. ^{1}H spectrum of compound **3d**[24] in $CDCl_3$.

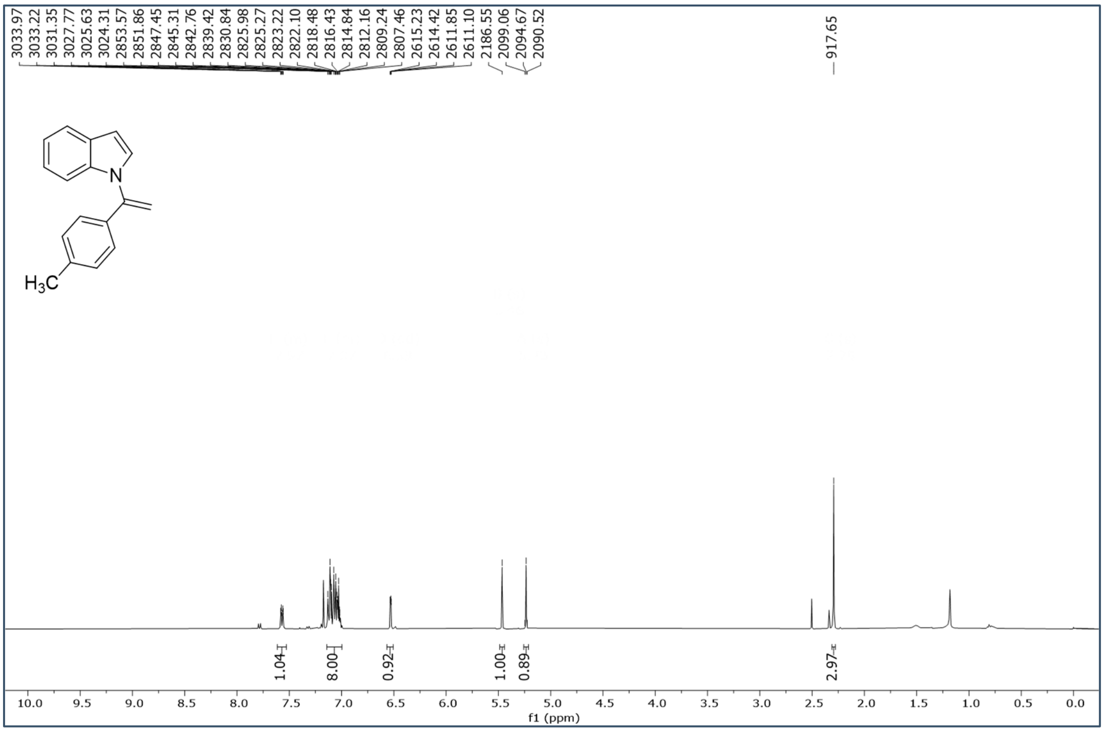

Figure S5. ^{1}H spectrum of compound **3e** in $CDCl_3$.

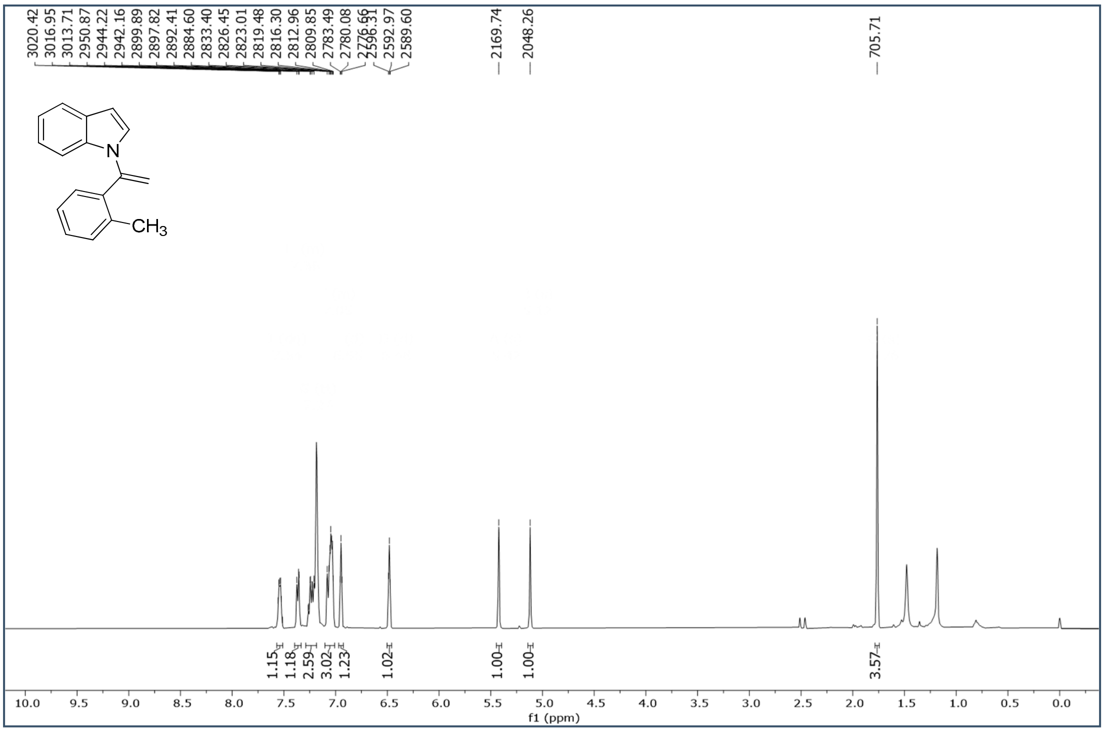

Figure S6. ^{1}H spectrum of compound **3f**[24] in $CDCl_3$.

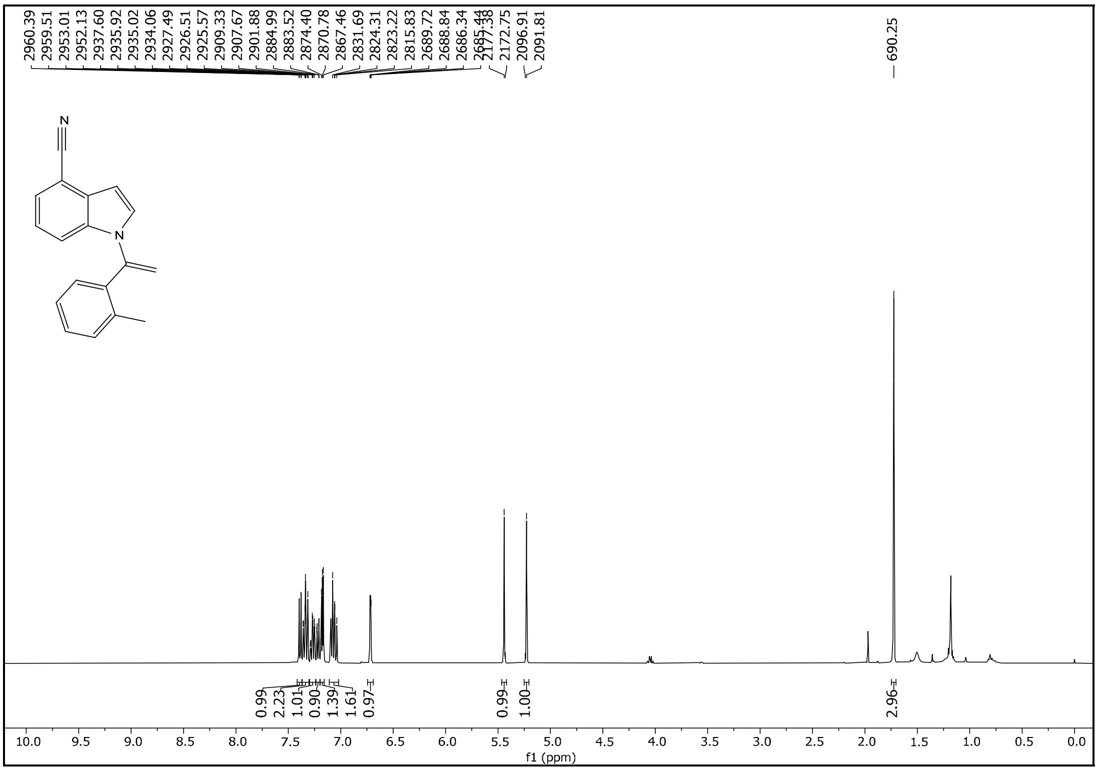

Figure S7. ^{1}H spectrum of compound **3g** in $CDCl_3$.

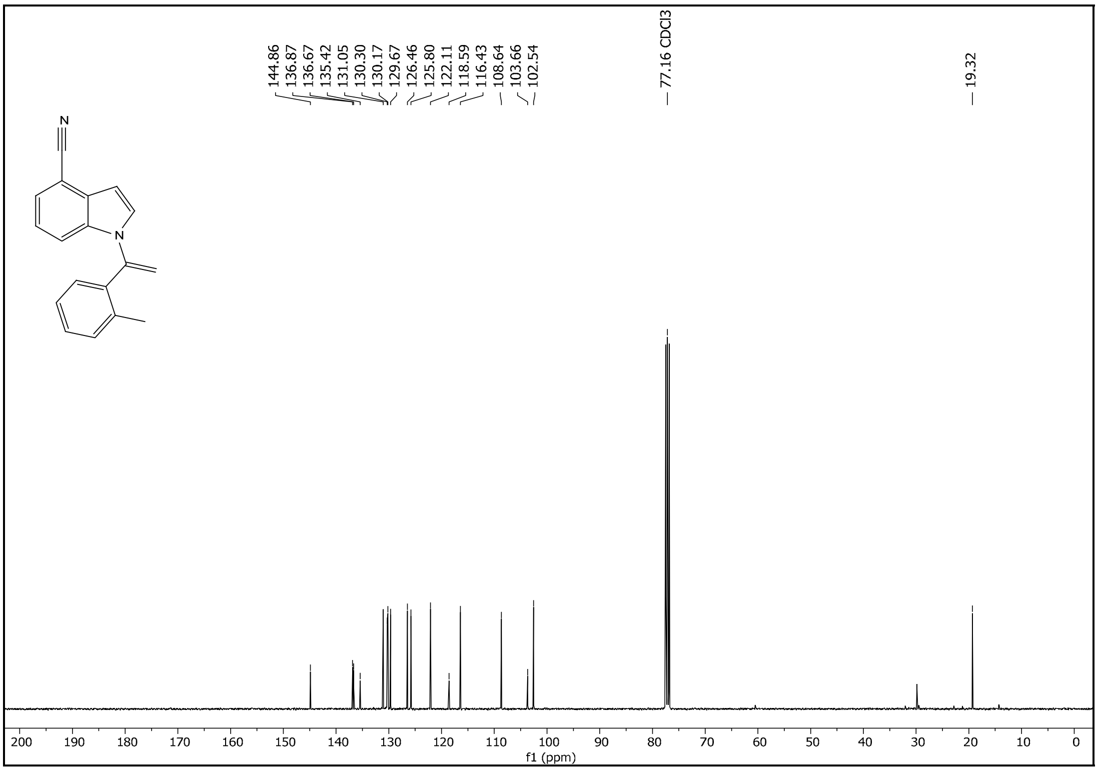

Figure S8. ^{13}C spectrum of compound **3g** in $CDCl_3$.

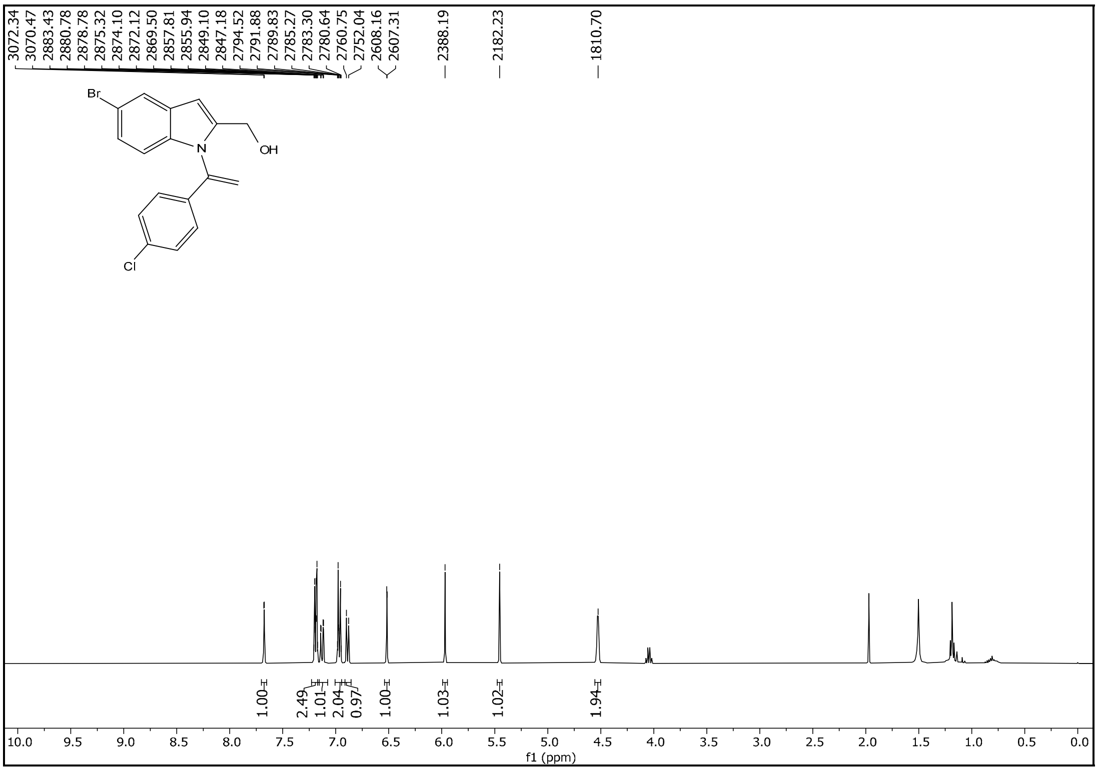

Figure S9. ^{1}H spectrum of compound **3h** in $CDCl_3$.

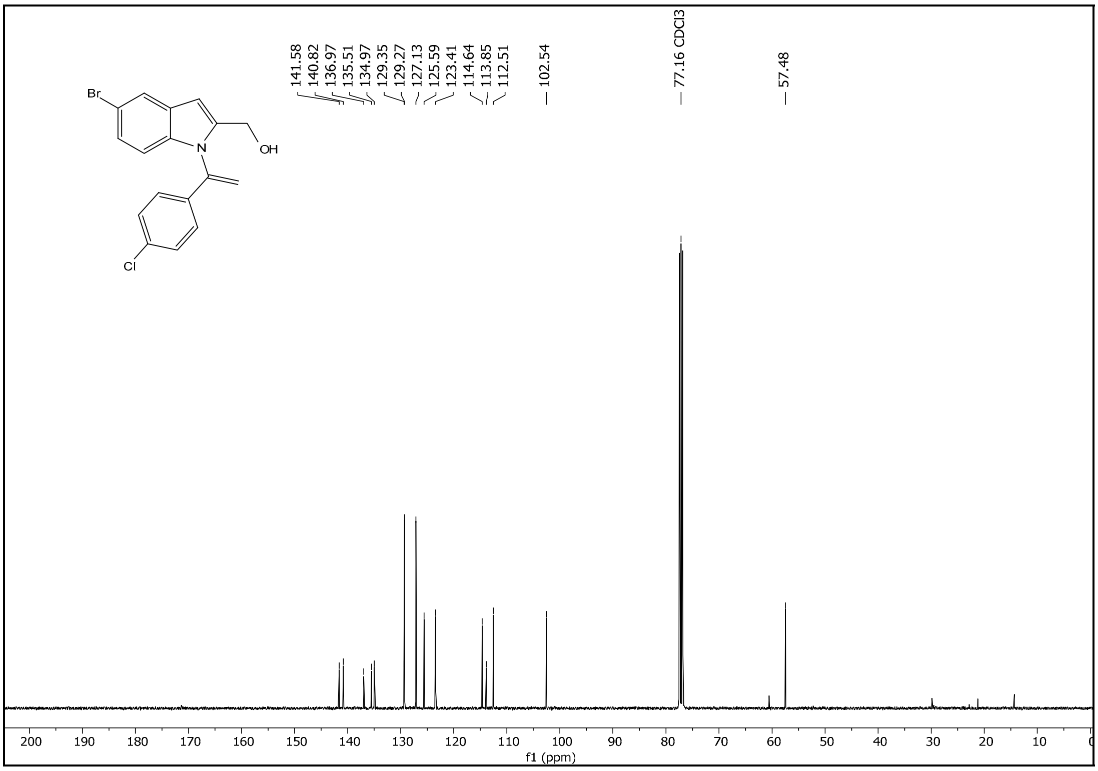

Figure S10. ^{13}C spectrum of compound **3h** in $CDCl_3$.

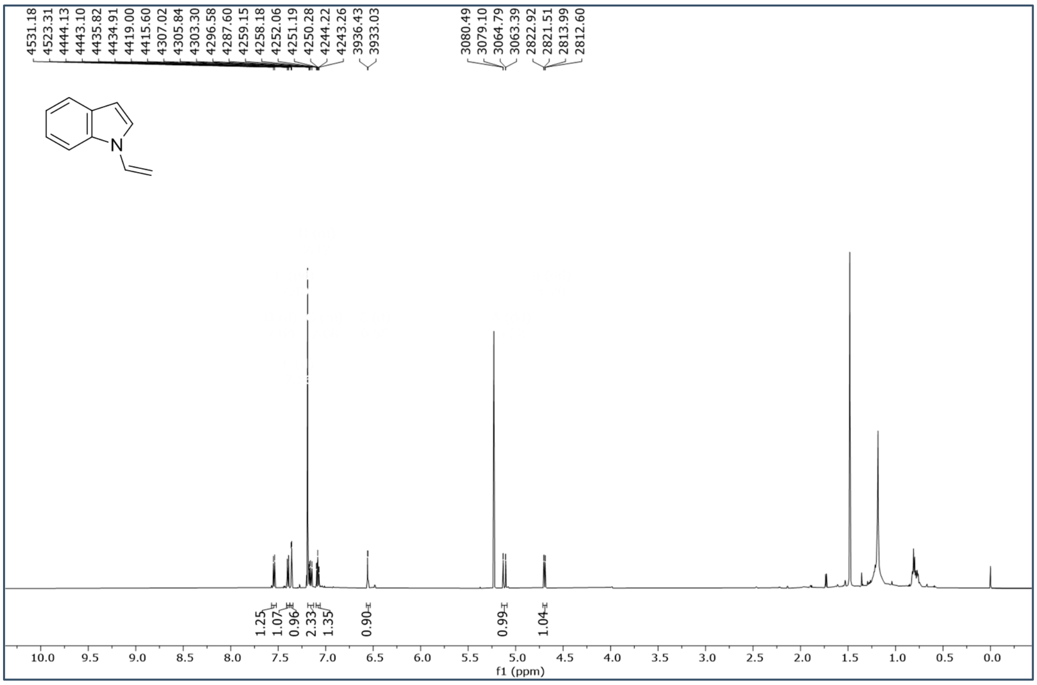

Figure S11. ^{1}H spectrum of compound **3i**[9] in $CDCl_3$.

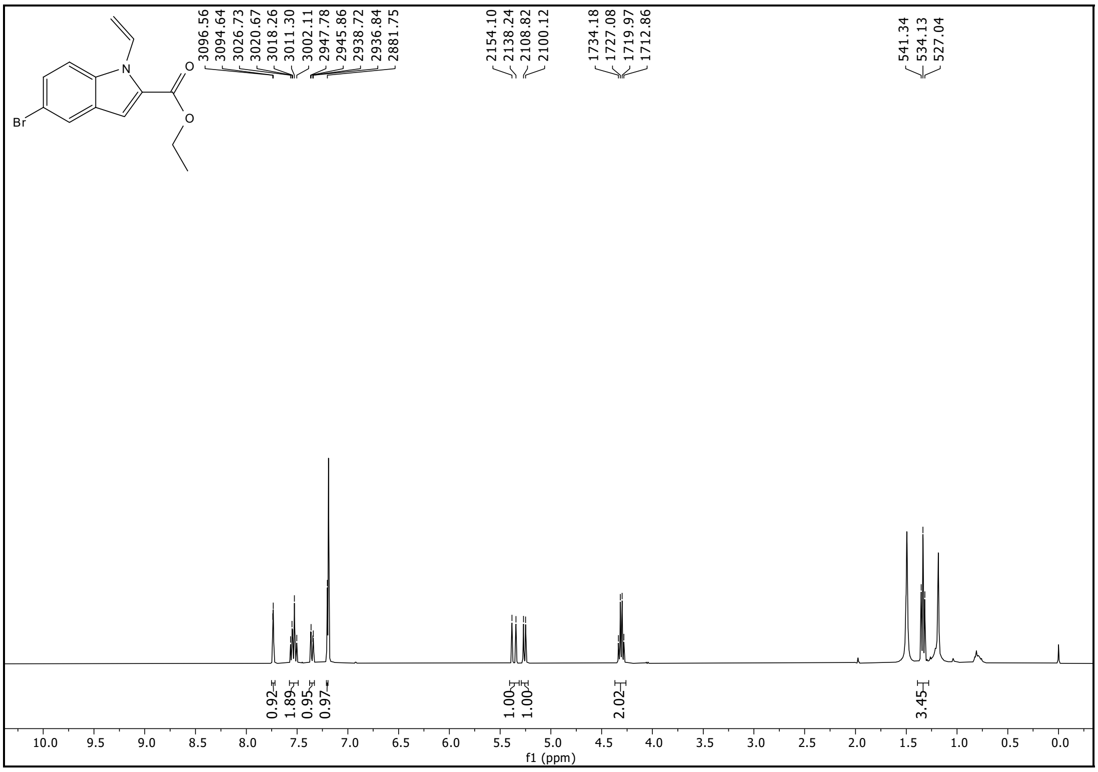

Figure S12. ^{1}H spectrum of compound **3j** in $CDCl_3$.

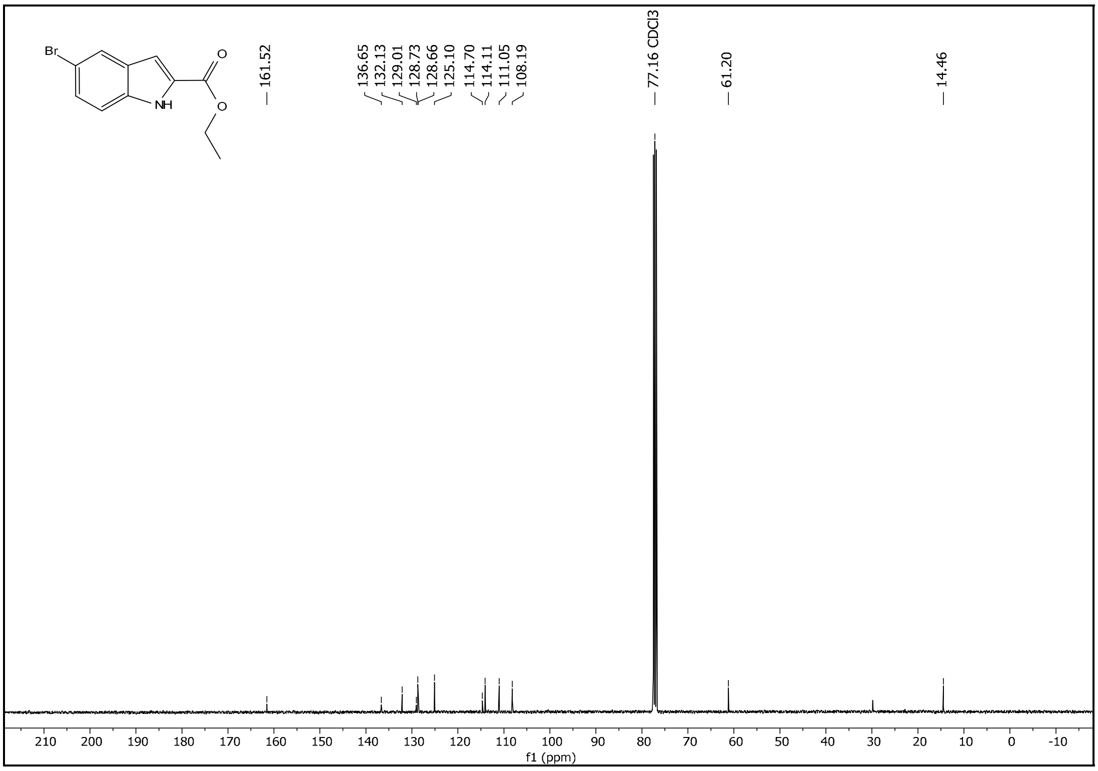

Figure S13. ^{13}C spectrum of compound **3j** in $CDCl_3$.

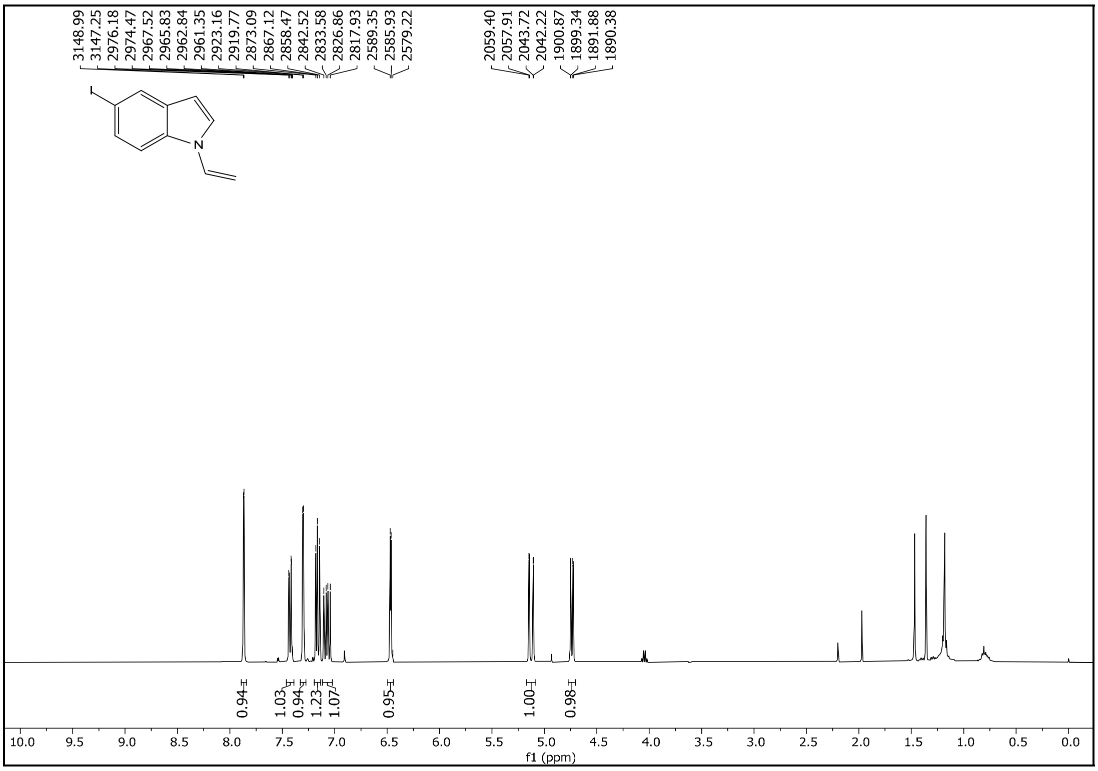

Figure S14. ^{1}H spectrum of compound **3k** in $CDCl_3$.

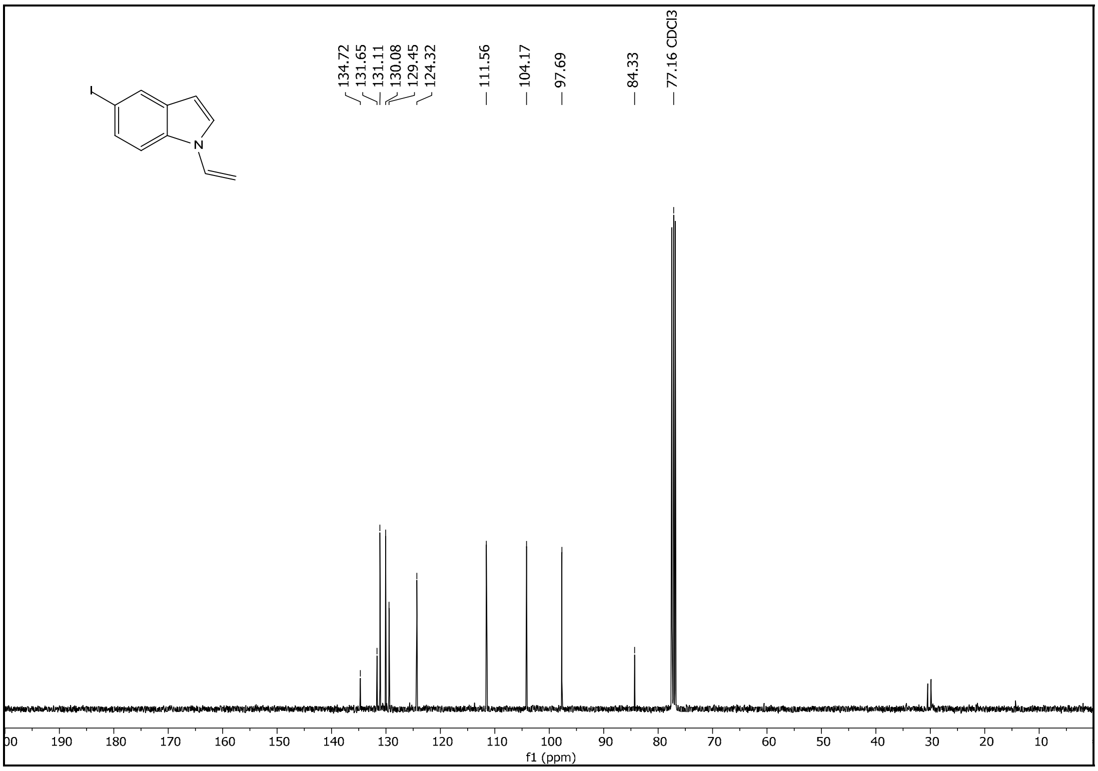

Figure S15. ^{13}C spectrum of compound **3k** in $CDCl_3$.

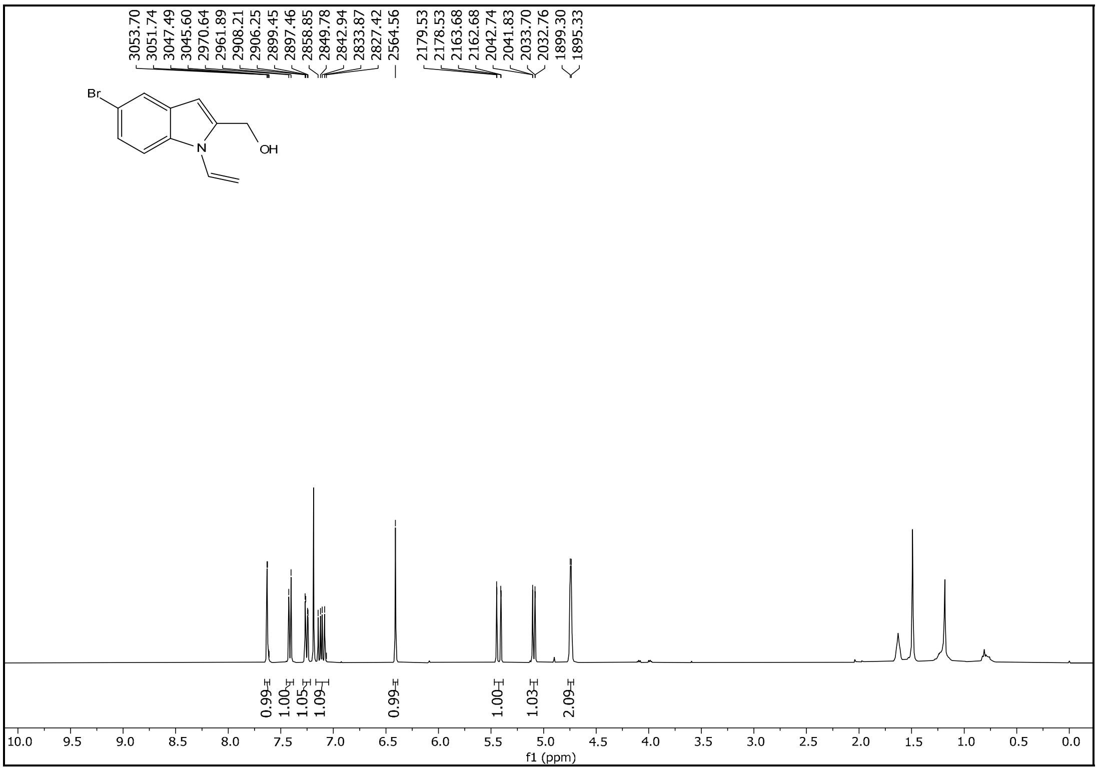

Figure S16. ^{1}H spectrum of compound **3l** in $CDCl_3$.

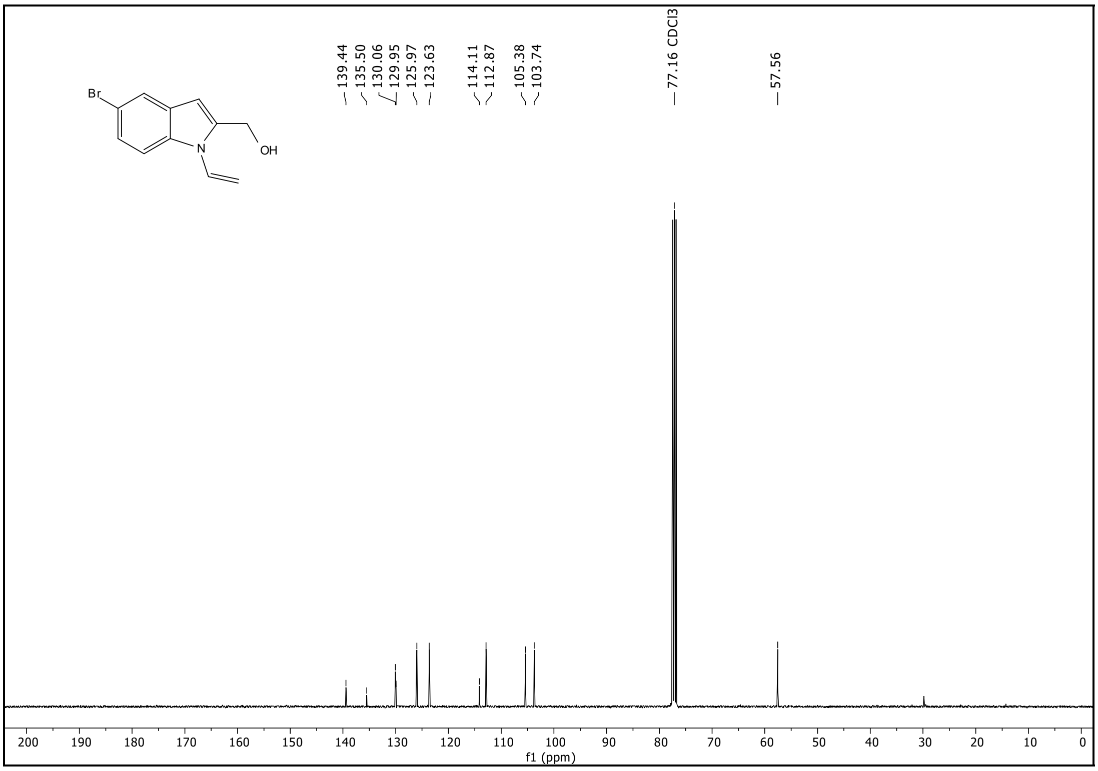

Figure S17. ^{13}C spectrum of compound **3l** in $CDCl_3$.

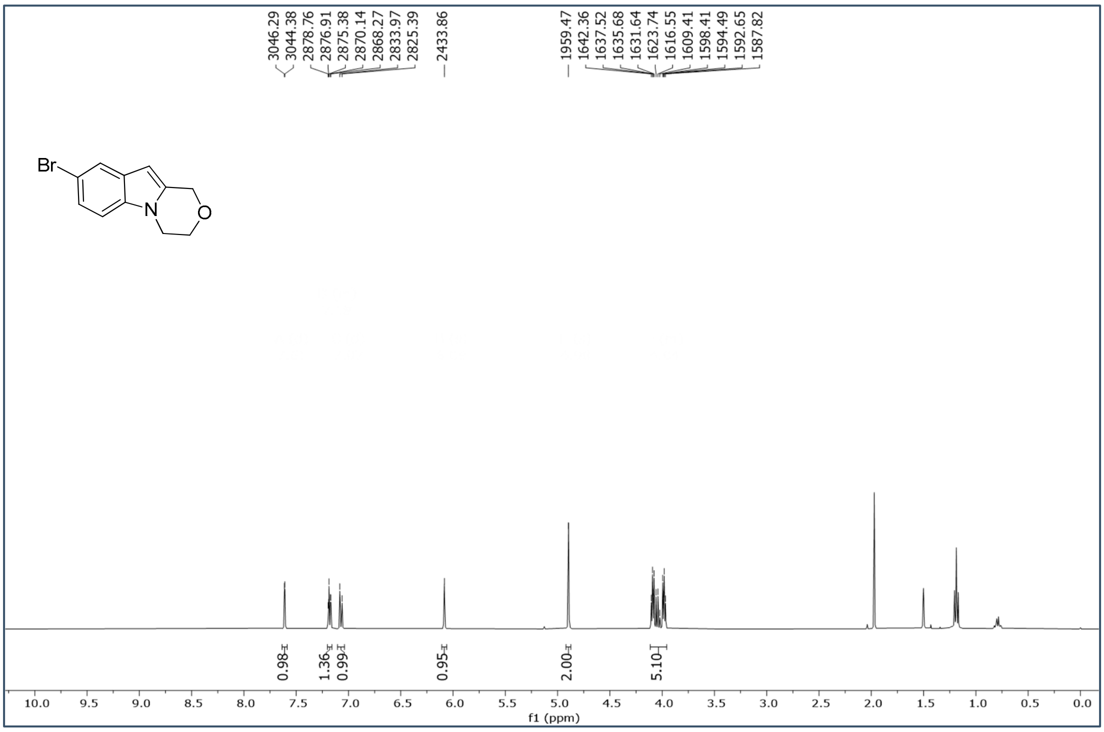

Figure S18. ^{1}H spectrum of 8-bromo-3, 4-dihydro-1*H*-[1,4]oxazino[4,3-*a*]indole[30] in $CDCl_3$.

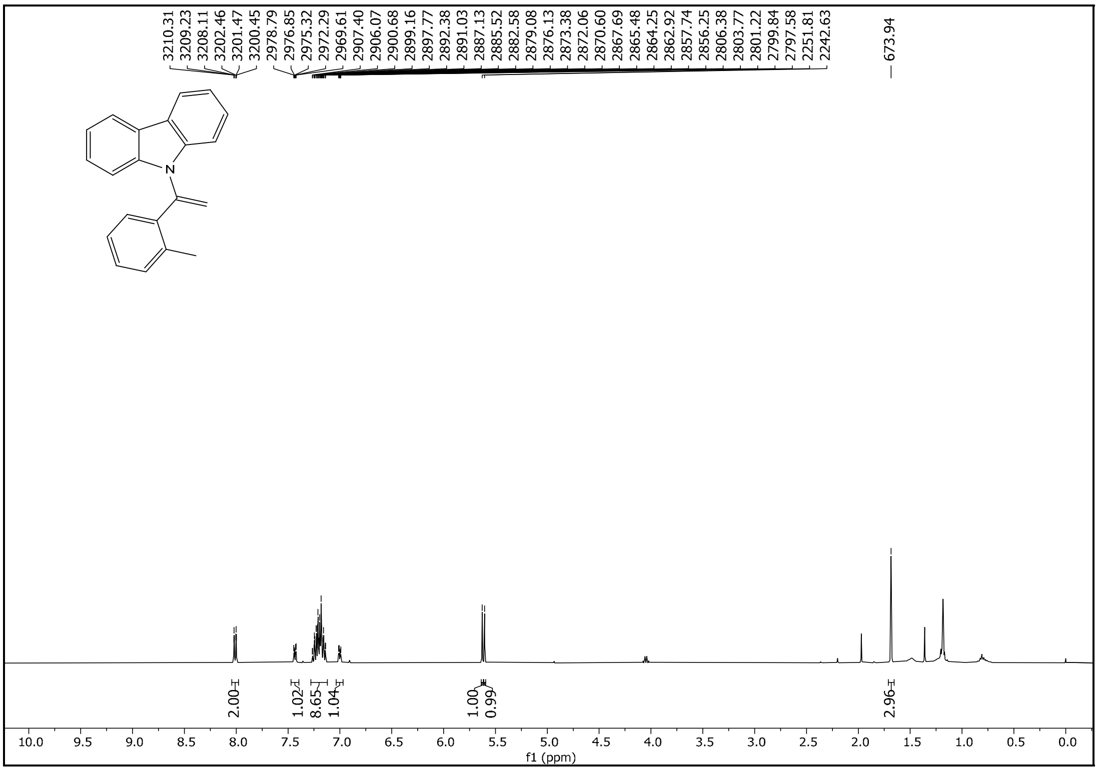

Figure S19. ^{1}H spectrum of compound **5a** in $CDCl_3$.

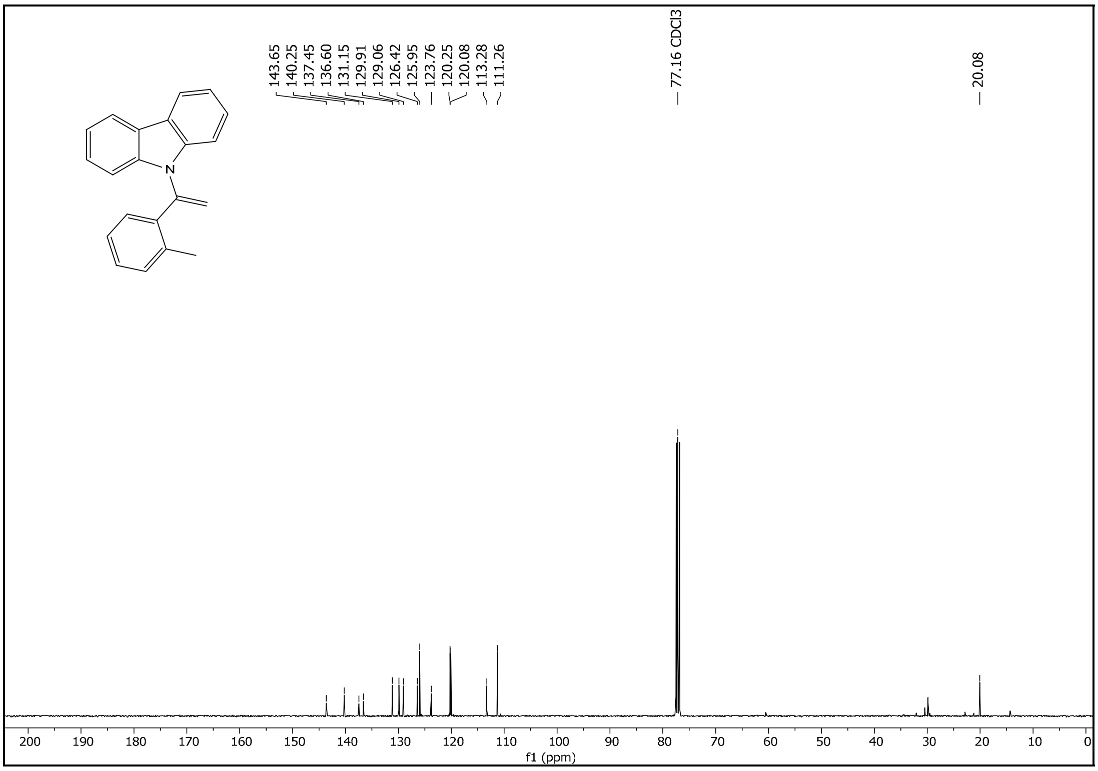

Figure S20. ^{13}C spectrum of compound **5a** in $CDCl_3$.

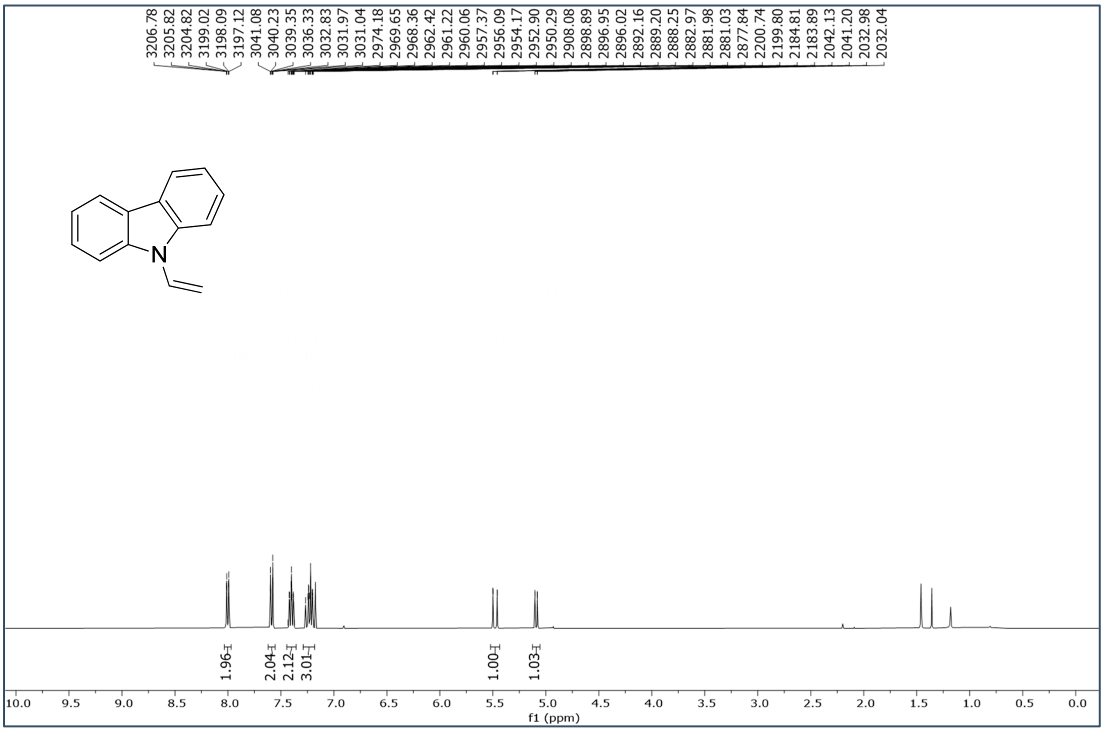

Figure S21. ^{1}H spectrum of compound **5b**[19] in $CDCl_3$.

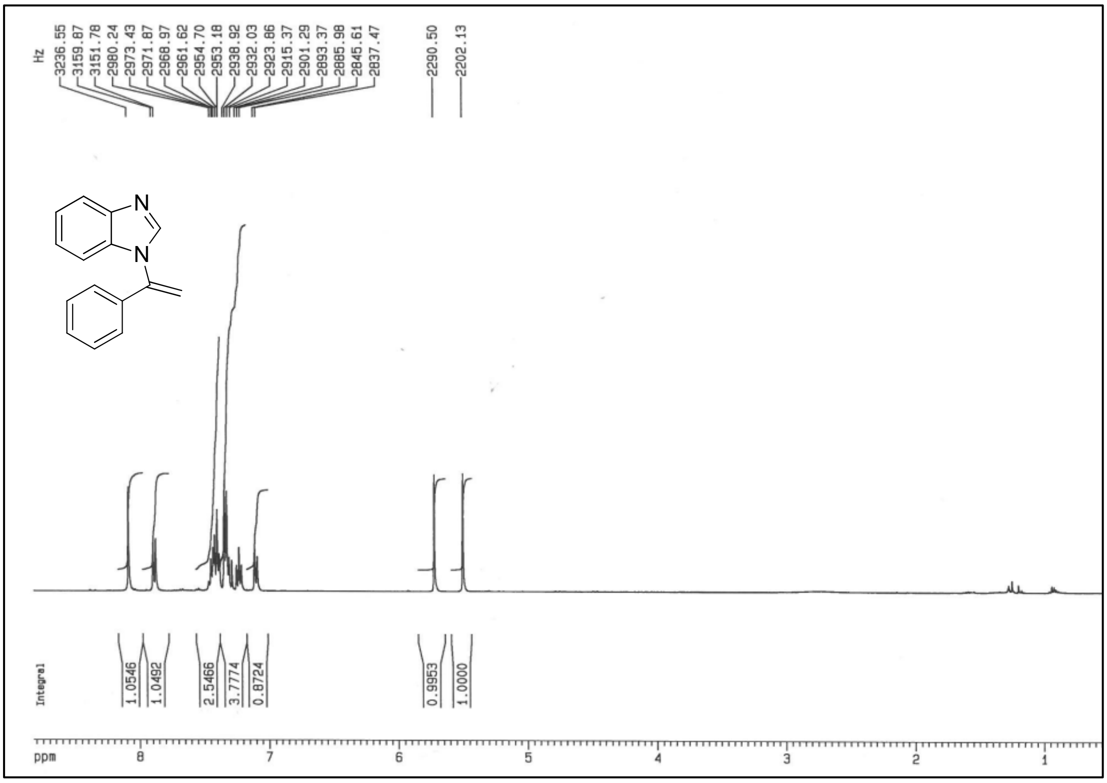

Figure S22. ^{1}H spectrum of compound **5c**[24] in $CDCl_3$.

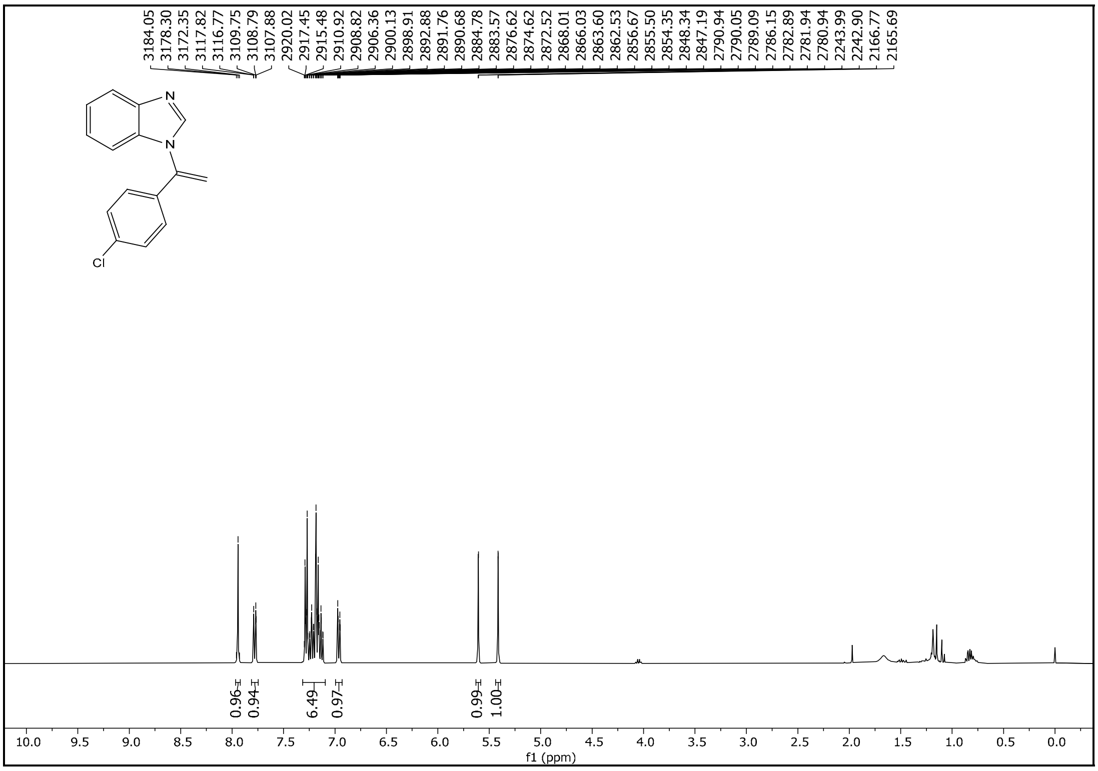

Figure S23. ^{1}H spectrum of compound **5d** in $CDCl_3$.

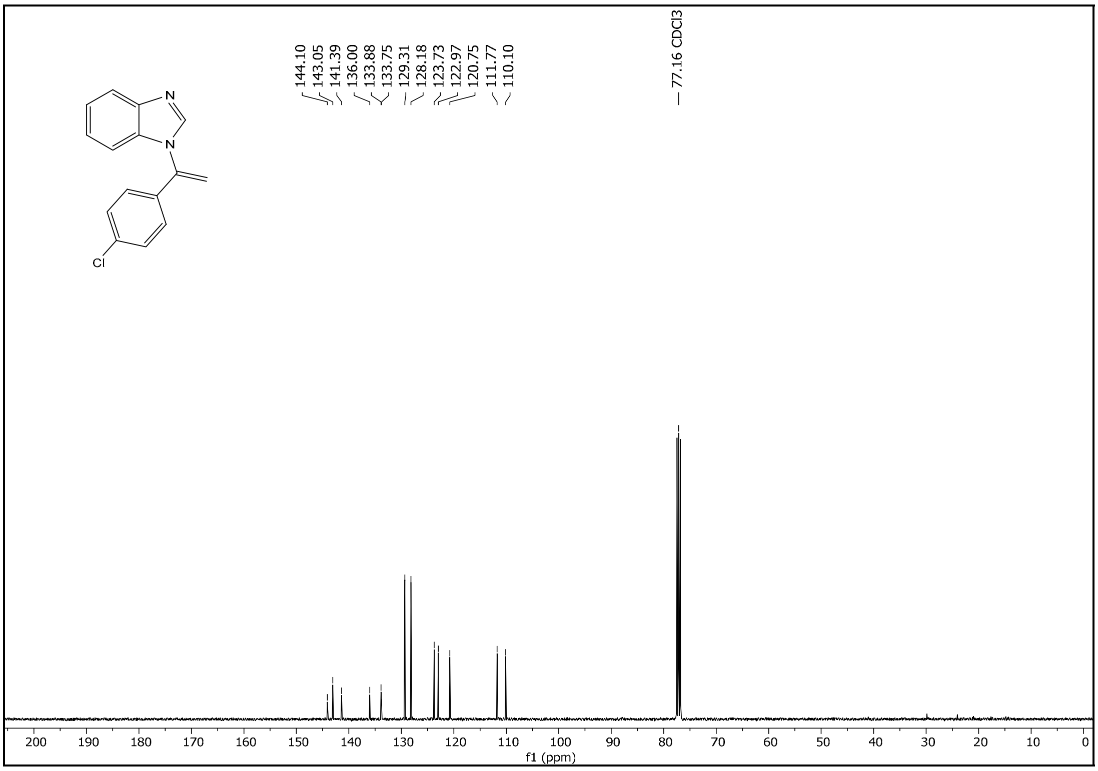

Figure S24. ^{13}C spectrum of compound **5d** in $CDCl_3$.

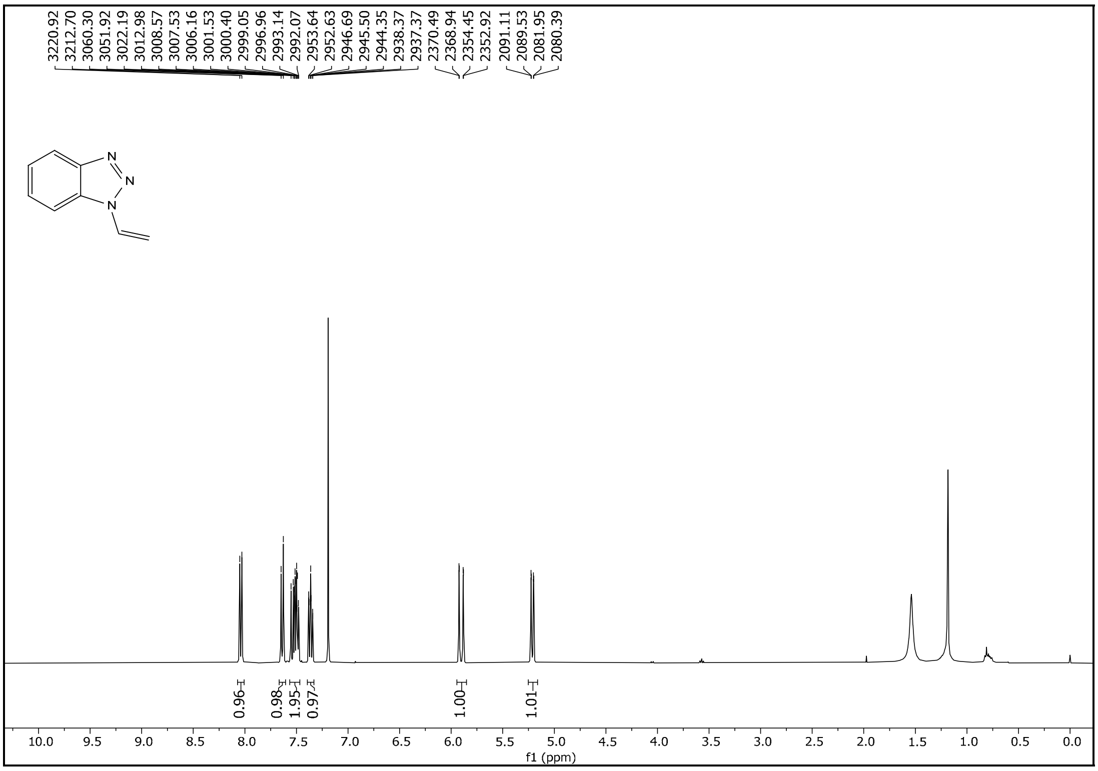

Figure S25. ^{1}H spectrum of compound **5e** in $CDCl_3$.

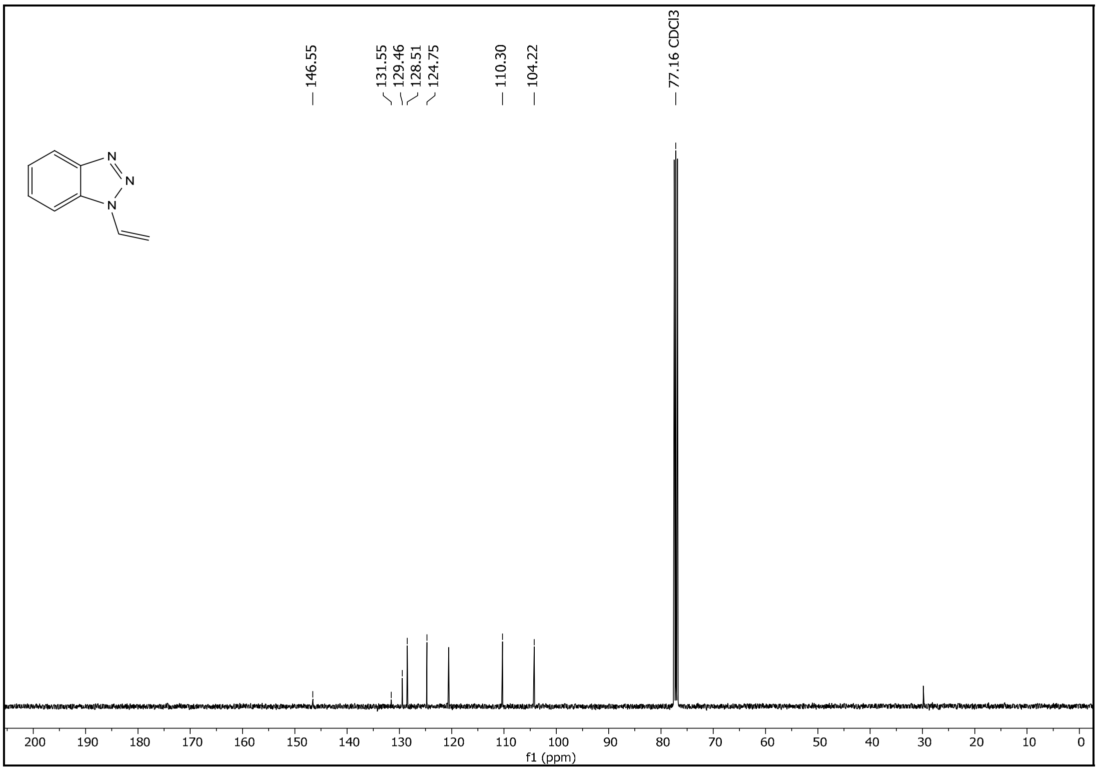

Figure S26. ^{13}C spectrum of compound **5e** in $CDCl_3$.

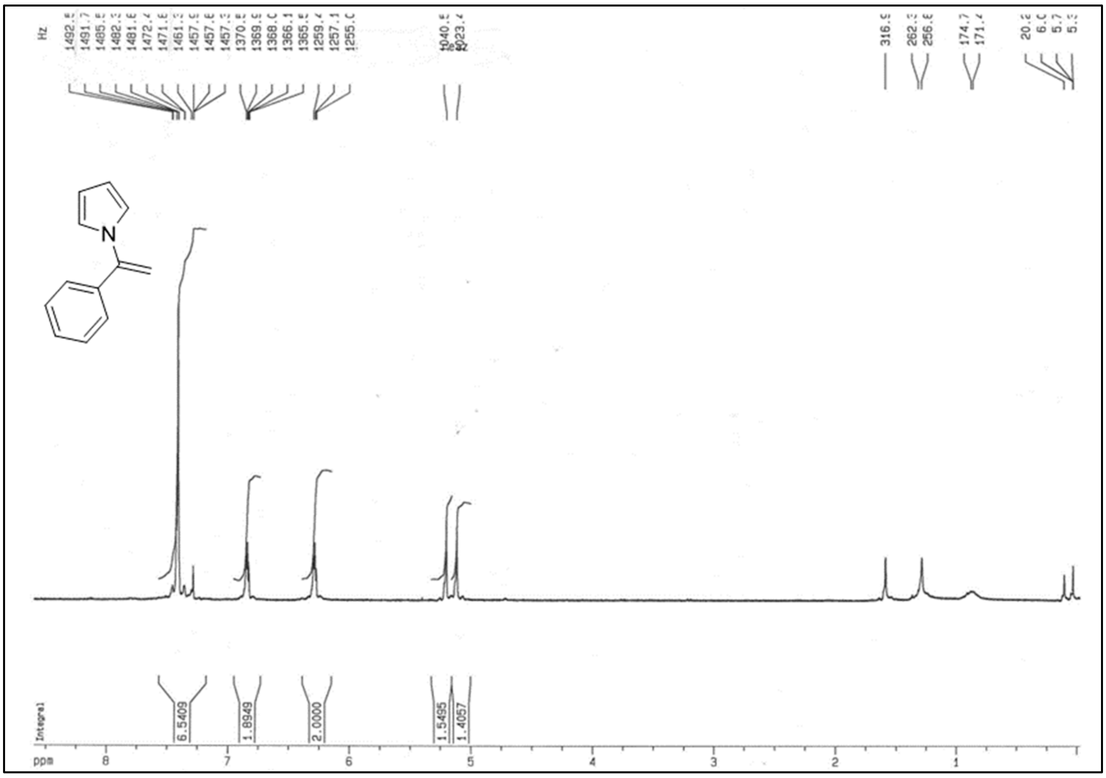

Figure S27. ^{1}H spectrum of compound **5f**[14] in $CDCl_3$.

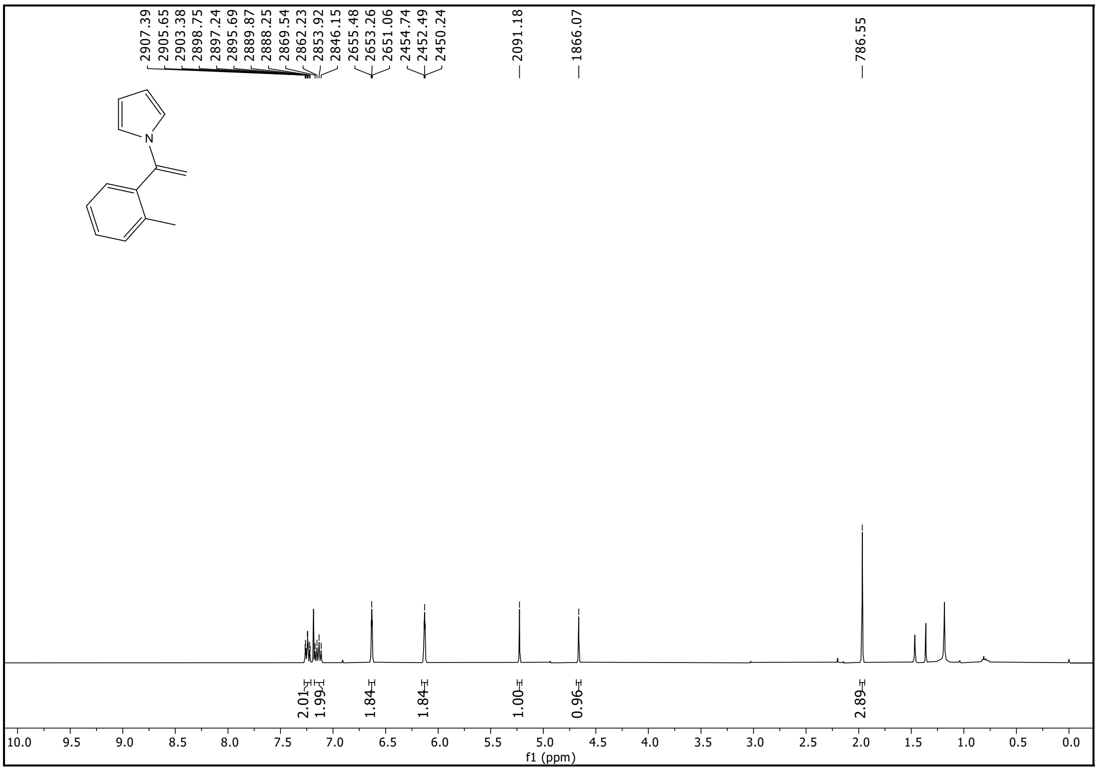

Figure S28. ^{1}H spectrum of compound **5g** in $CDCl_3$

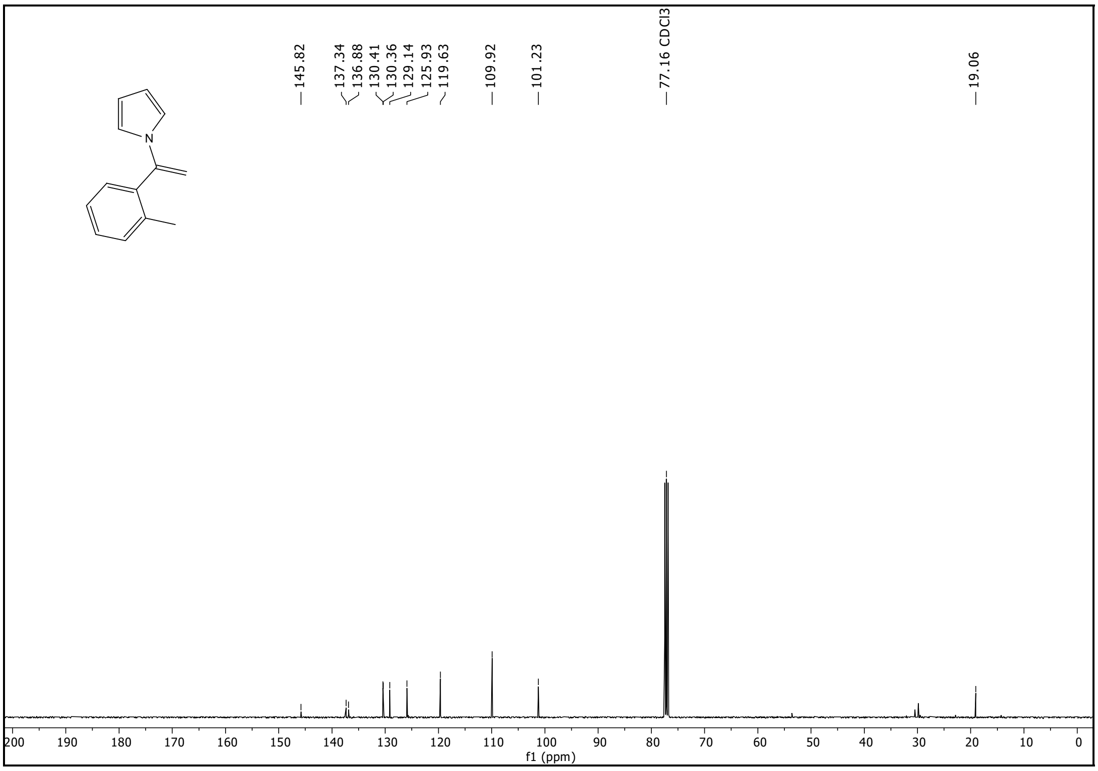

Figure S29. ^{1}H spectrum of compound **5g** in $CDCl_3$.

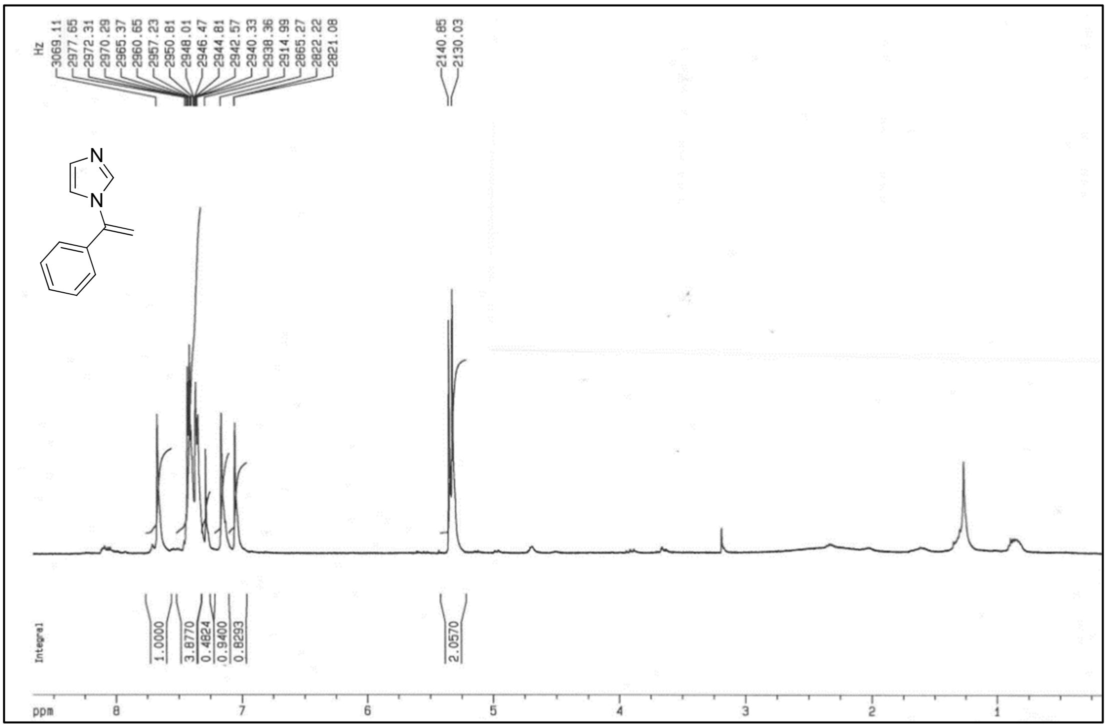

Figure S30. ^{1}H spectrum of compound **5h**[14] in $CDCl_3$.

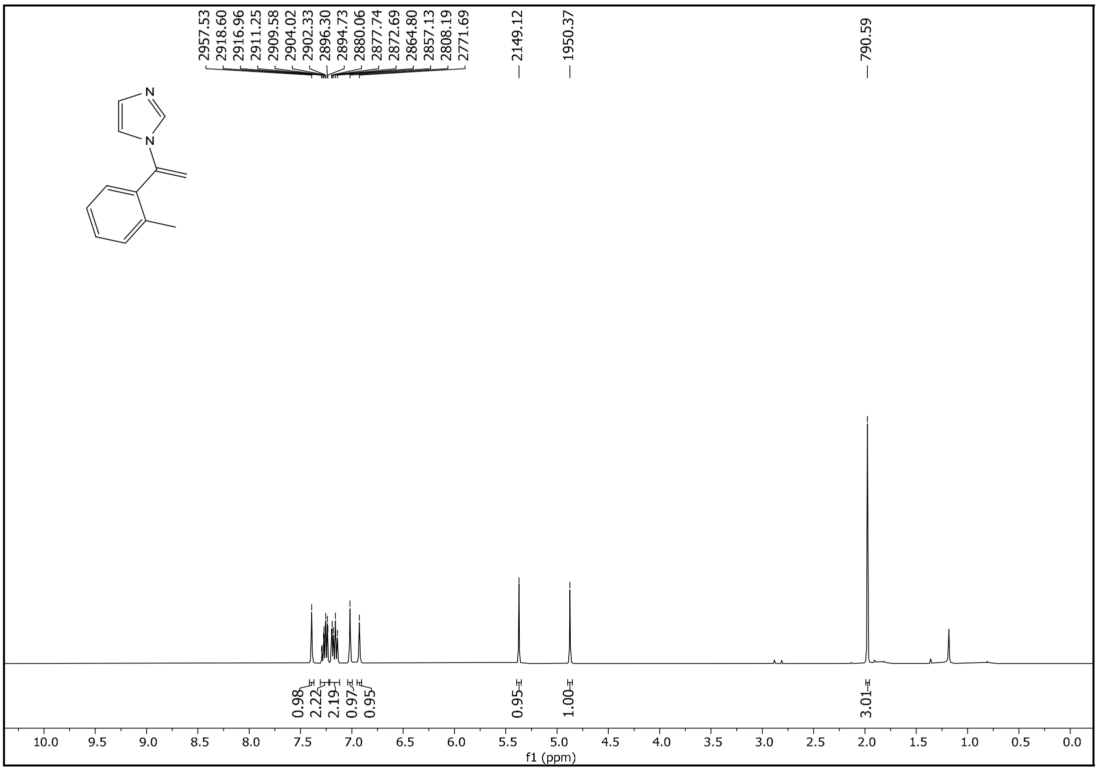

Figure S31. ^{1}H spectrum of compound **5i** in $CDCl_3$.

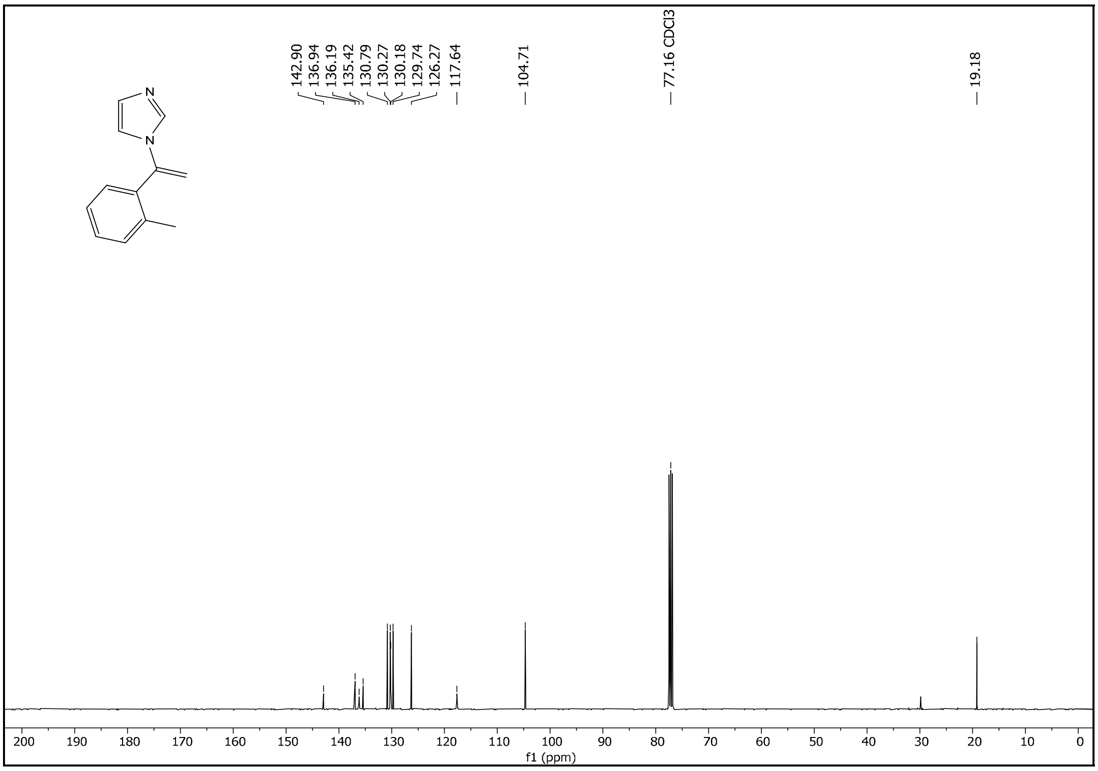

Figure S32. ^{13}C spectrum of compound **5i** in $CDCl_3$.

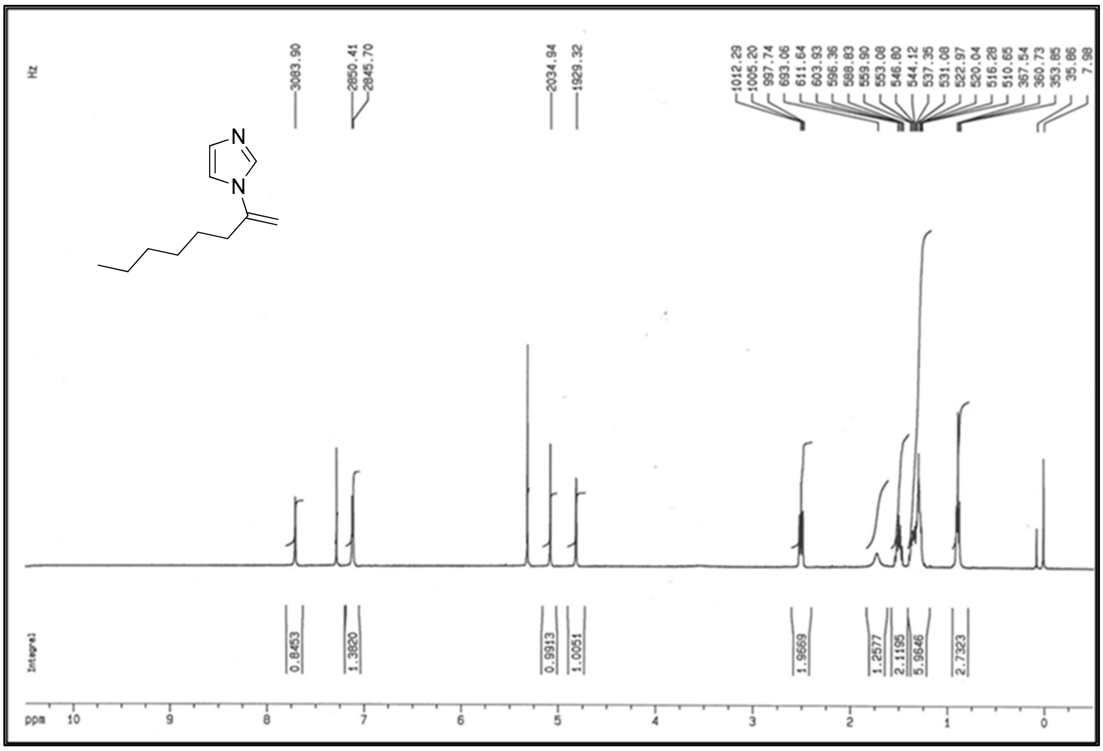

Figure S33. ^{1}H spectrum of compound **5j** in $CDCl_3$.

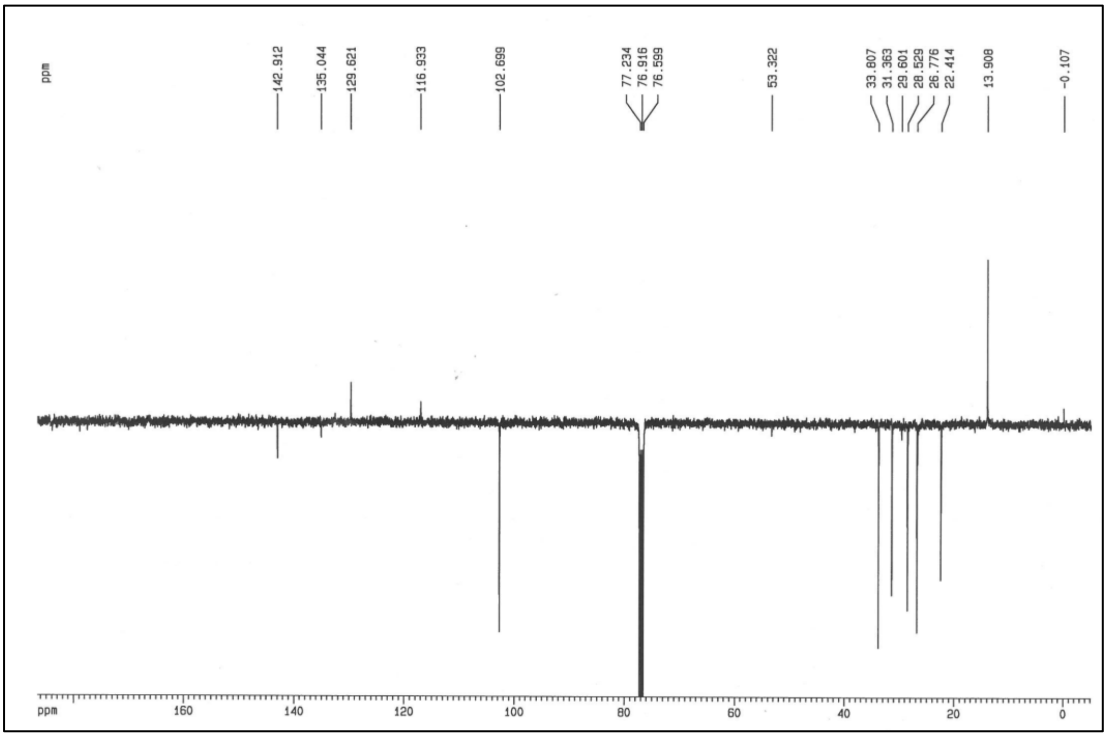

Figure S34. ^{13}C spectrum of compound **5j** in $CDCl_3$.

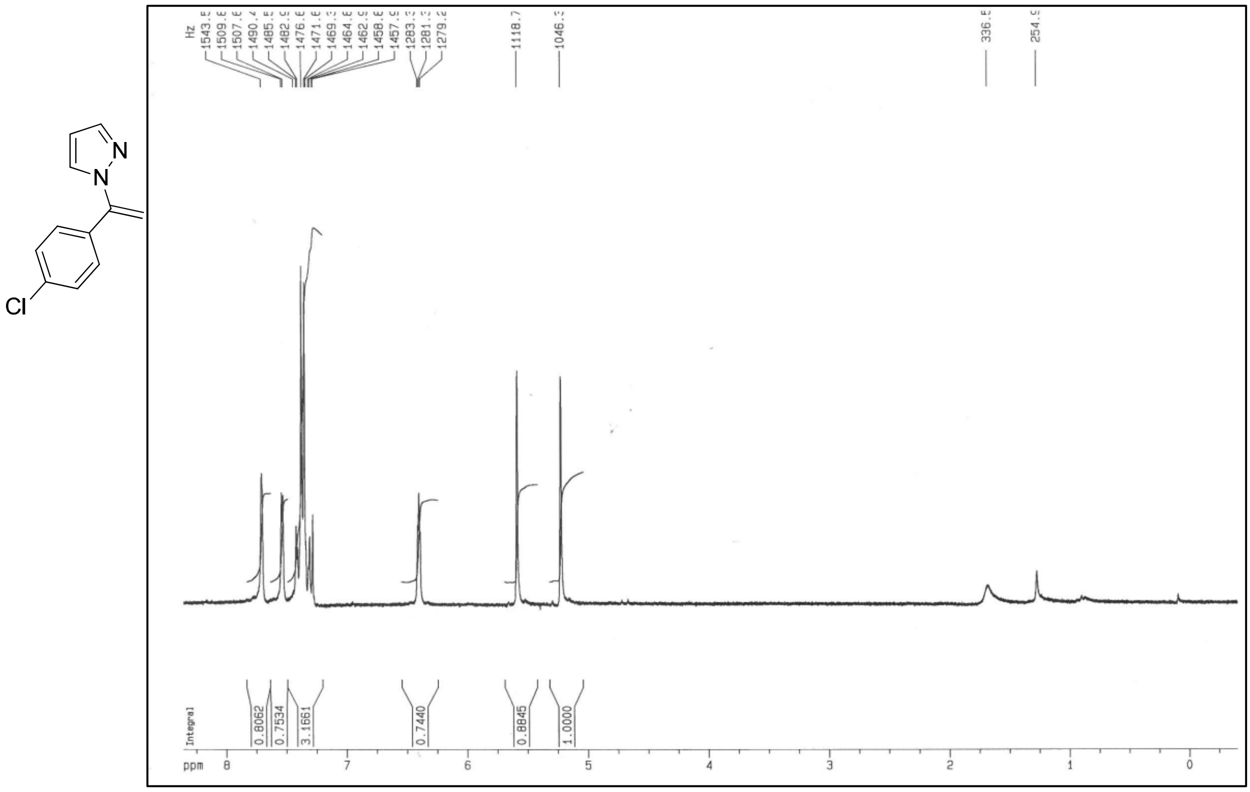

Figure S35. ^{1}H spectrum of compound **5k**[46] in $CDCl_3$.

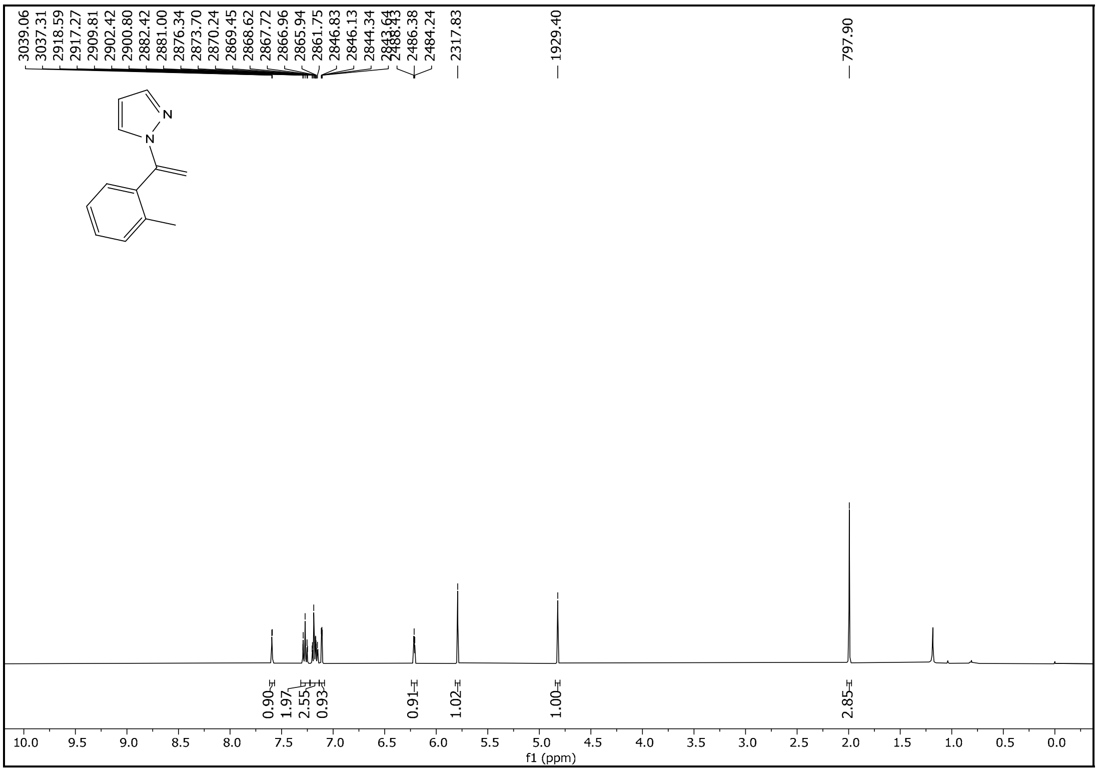

Figure S36. ^{1}H spectrum of compound **5l** in $CDCl_3$.

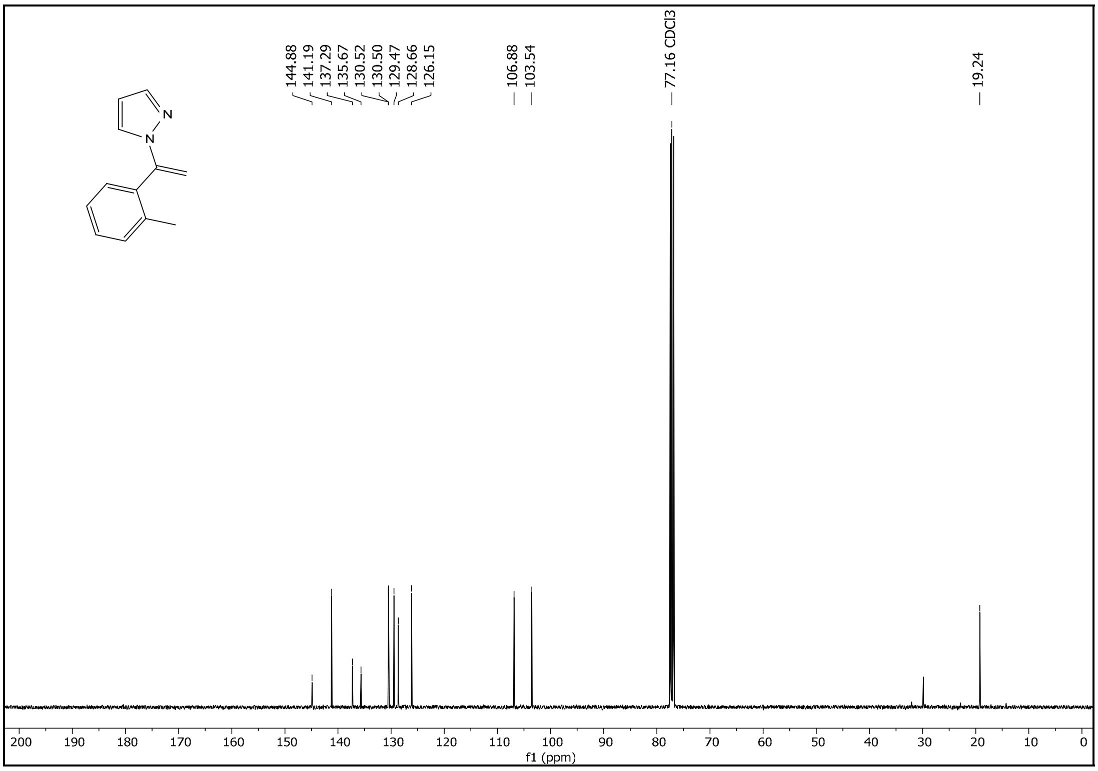

Figure S37. ^{13}C spectrum of compound **5l** in $CDCl_3$.

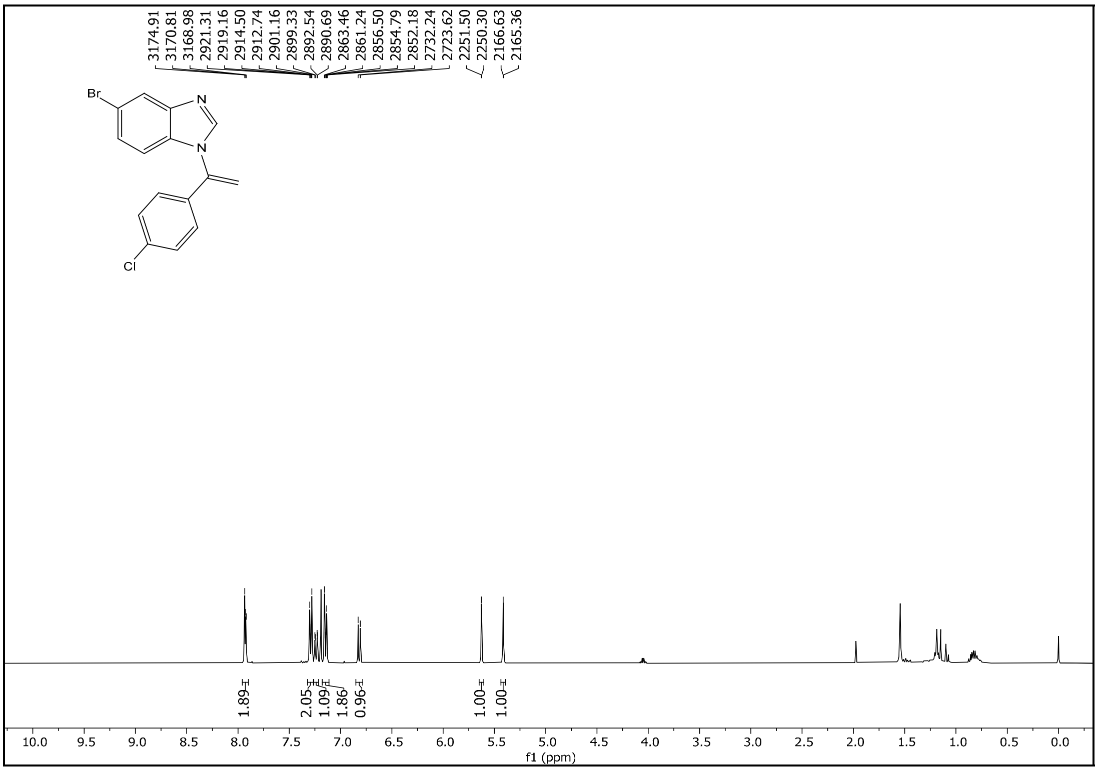

Figure S38. ^{1}H spectrum of compound **5m** in $CDCl_3$.

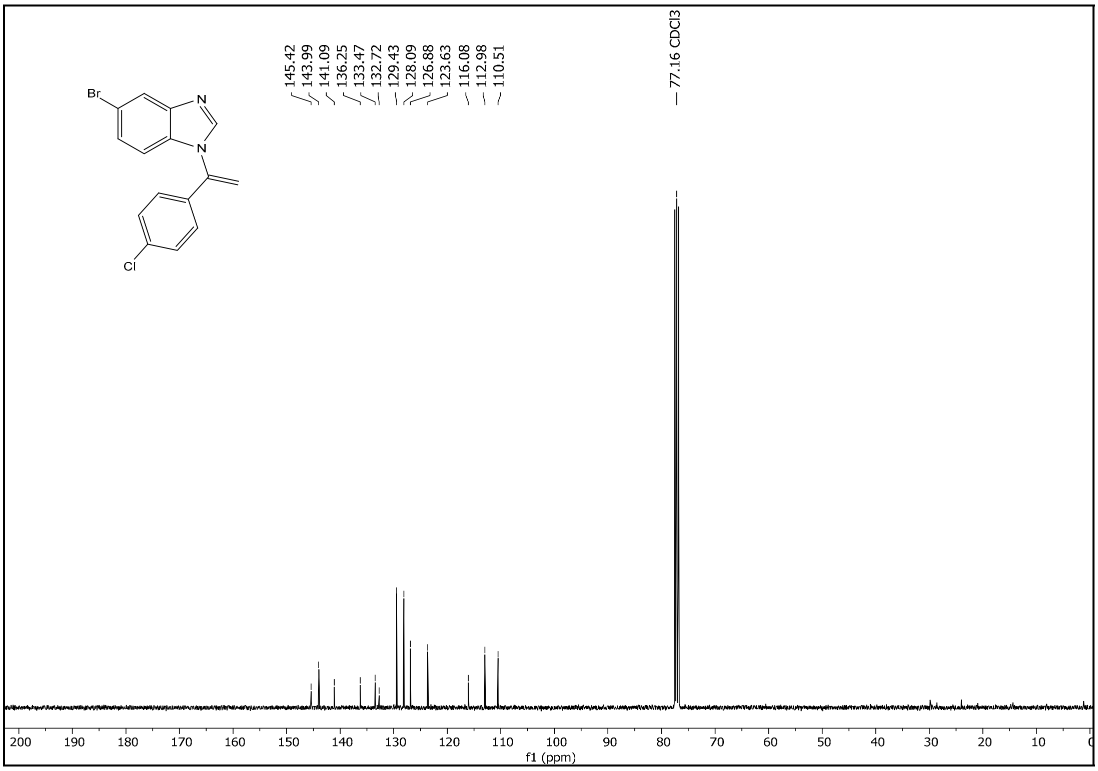

Figure S39. ^{13}C spectrum of compound **5m** in $CDCl_3$.

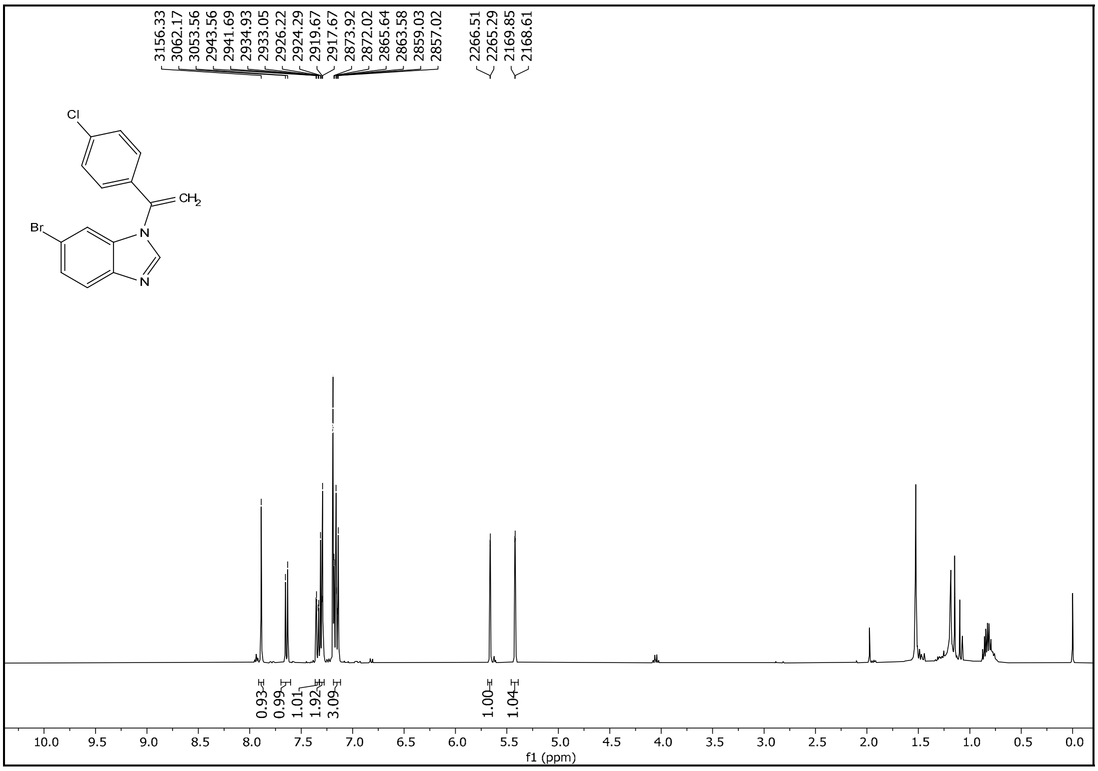

Figure S40. ^{1}H spectrum of compound **5m′** in $CDCl_3$.

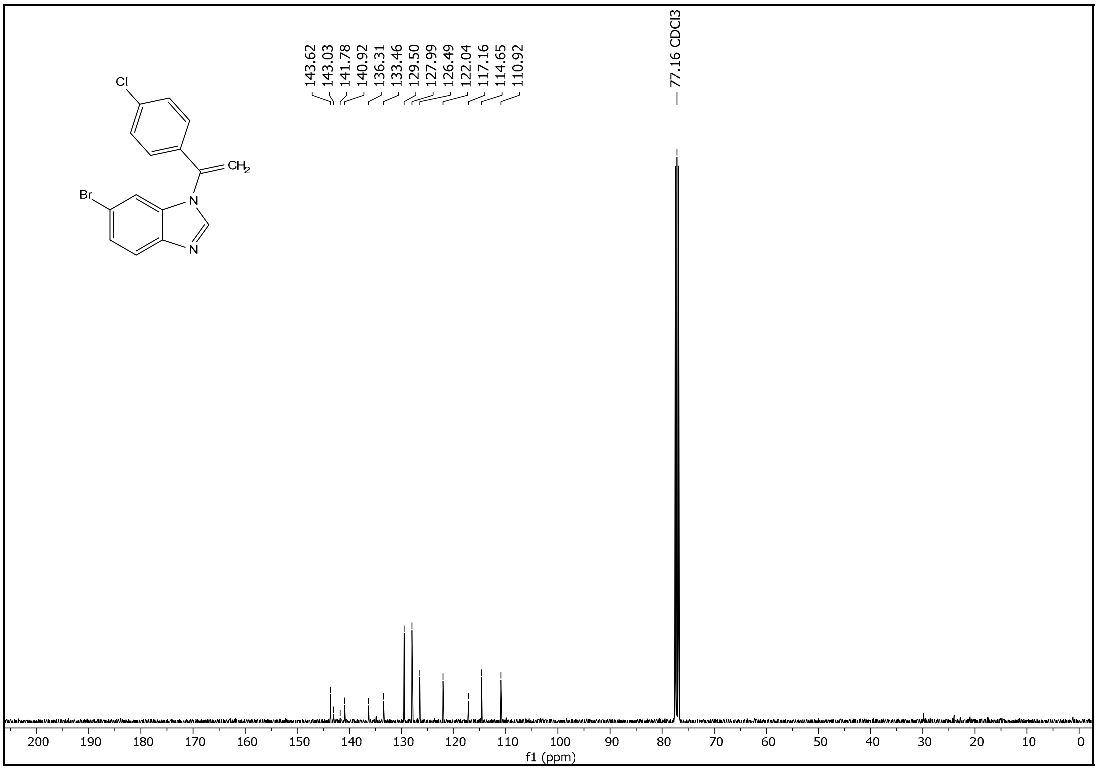

Figure S41. ^{13}C spectrum of compound **5m′** in $CDCl_3$.

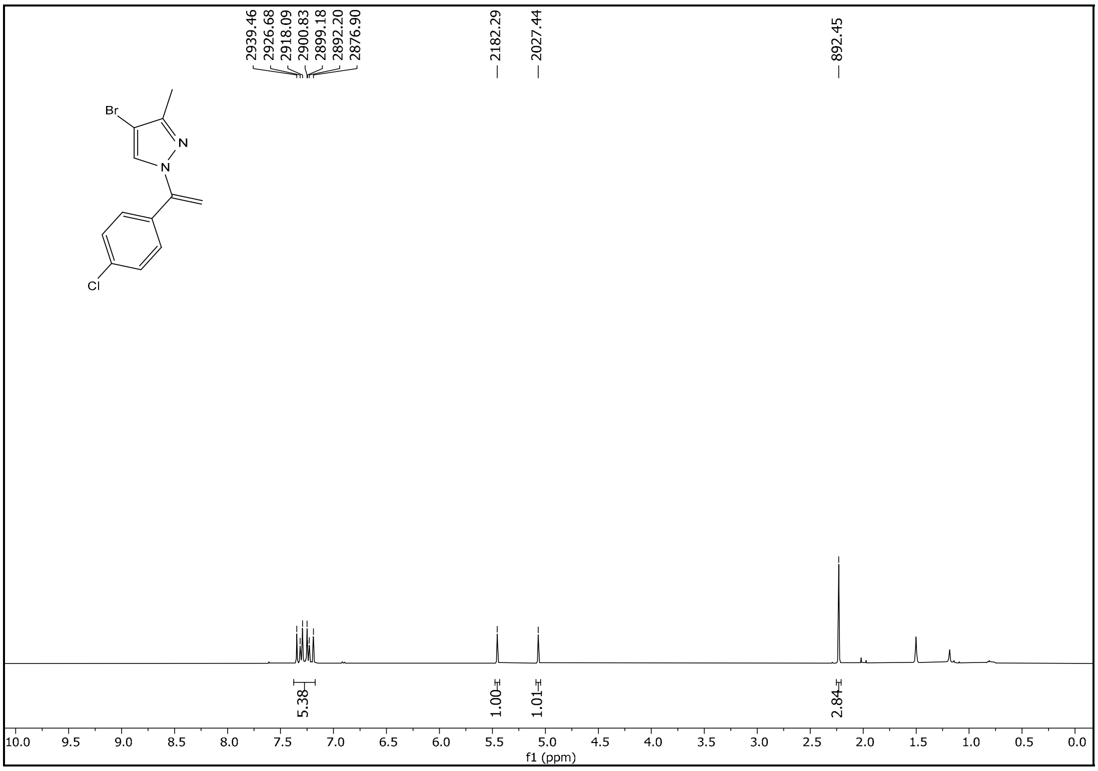

Figure S42. ^{1}H spectrum of compound **5n** in $CDCl_3$

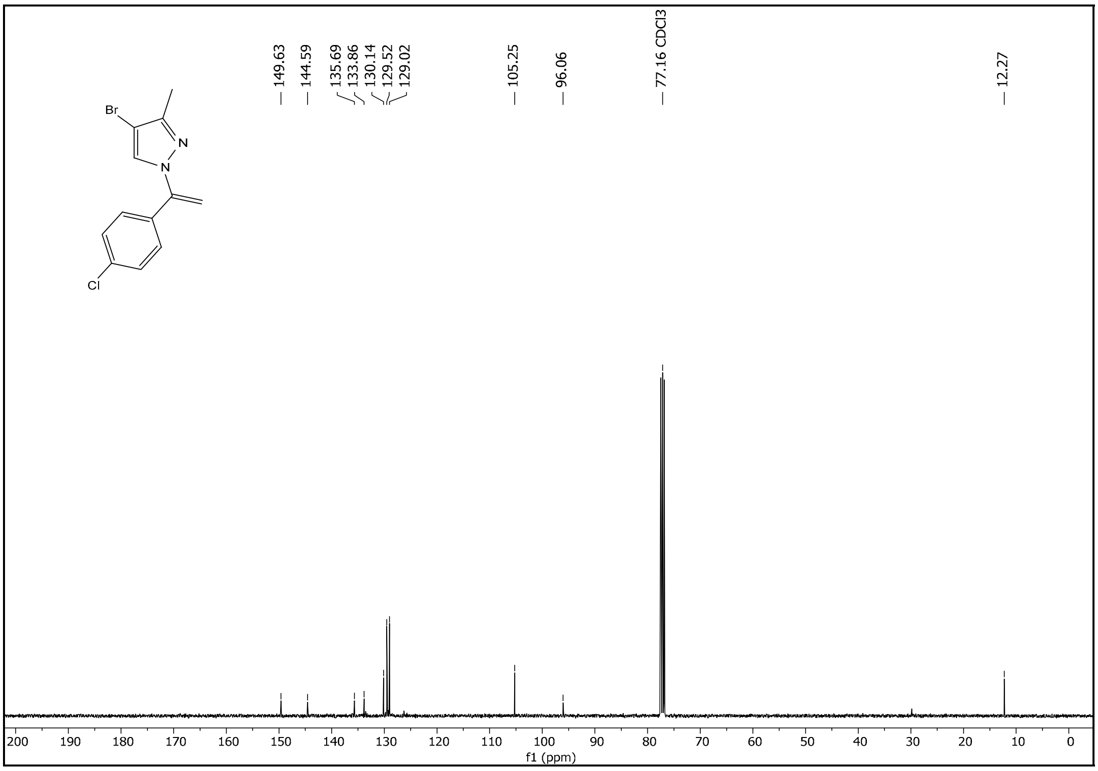

Figure S43. ^{13}C spectrum of compound **5n** in $CDCl_3$.

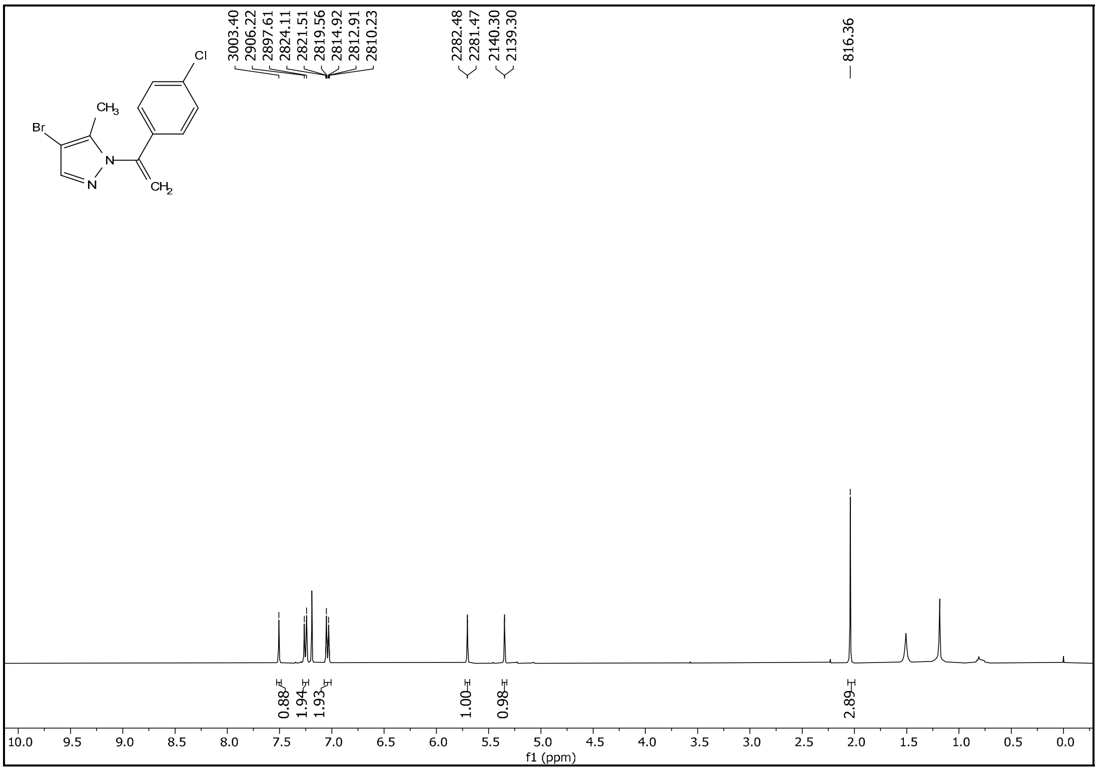

Figure S44. ^{1}H spectrum of compound **5n′** in $CDCl_3$.

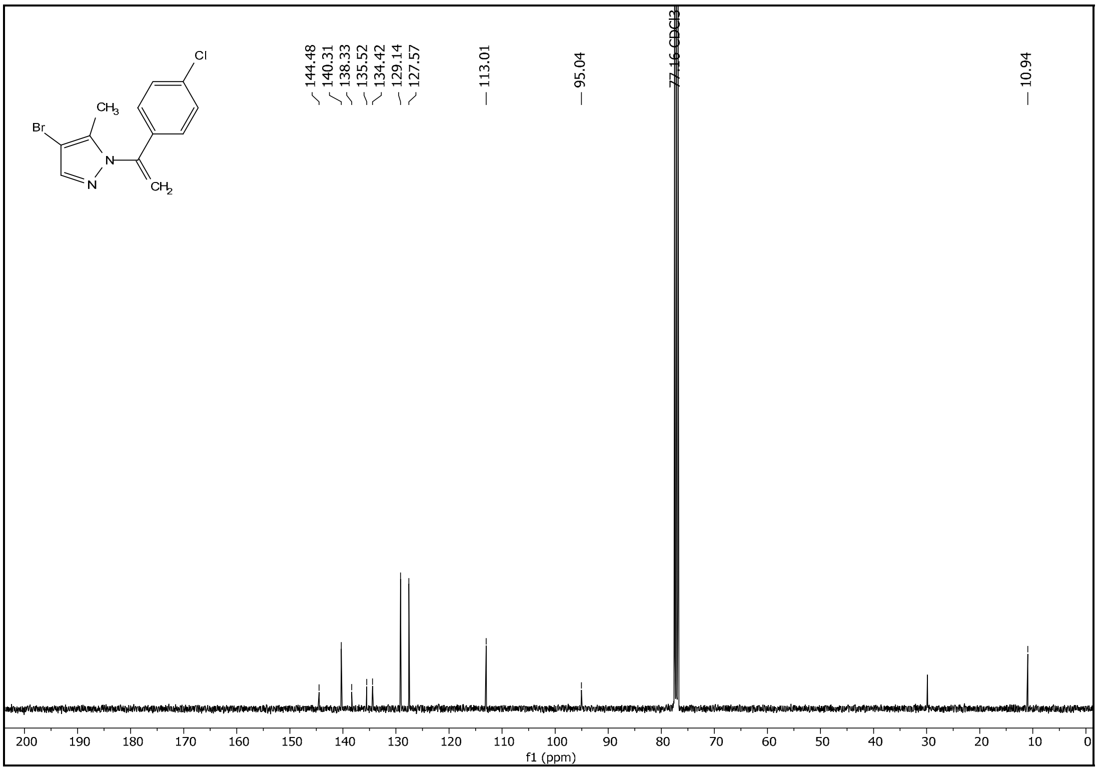

Figure S45. ^{13}C spectrum of compound **5n′** in $CDCl_3$.

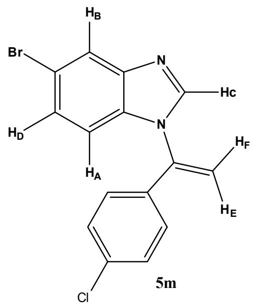

5m

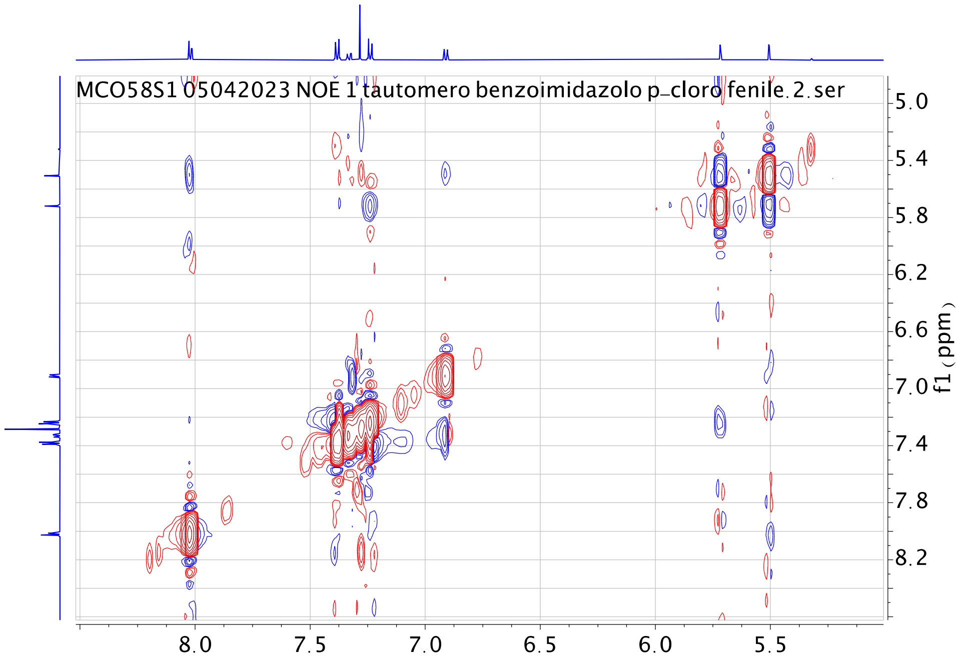

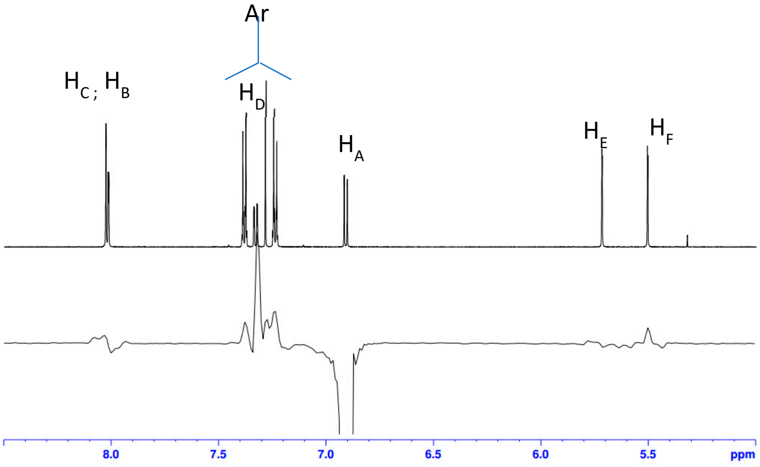

Figure S46. NOESY (600 MHz, $CDCl_3$) correlations of compound **5m.**

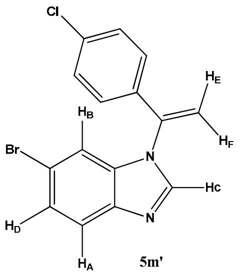

5m'

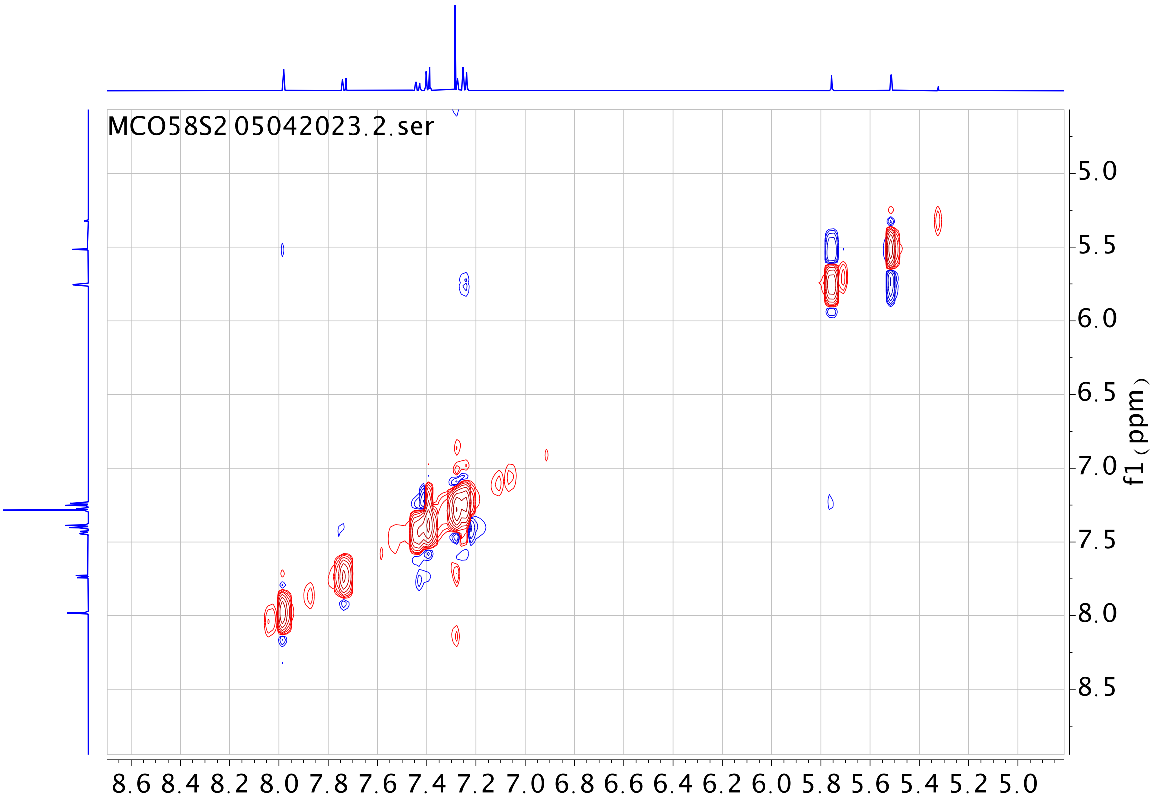

Figure S47. NOESY (600 MHz, CDCl3) correlations of compound **5m′.**

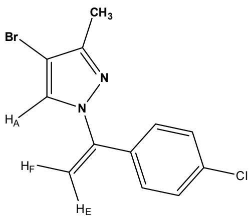

5n

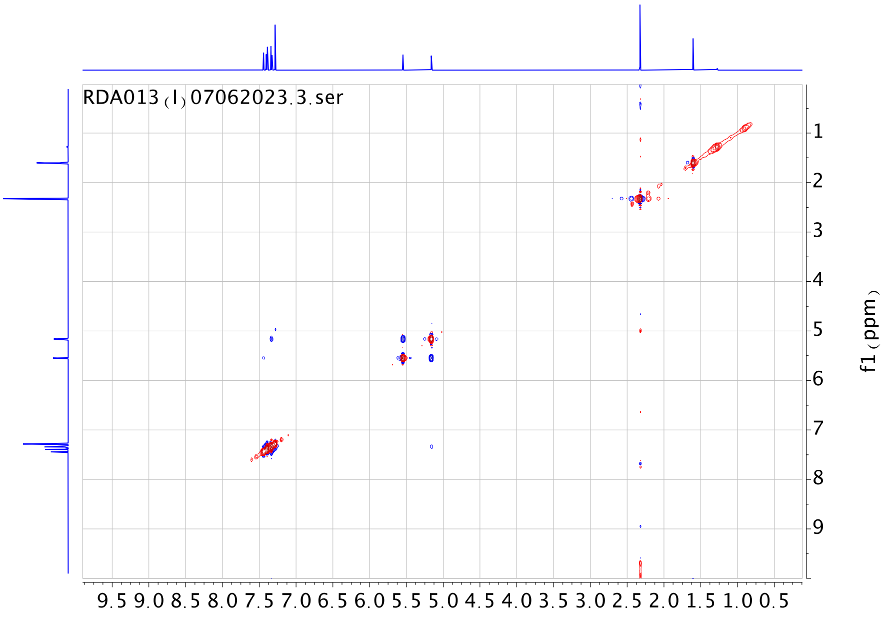

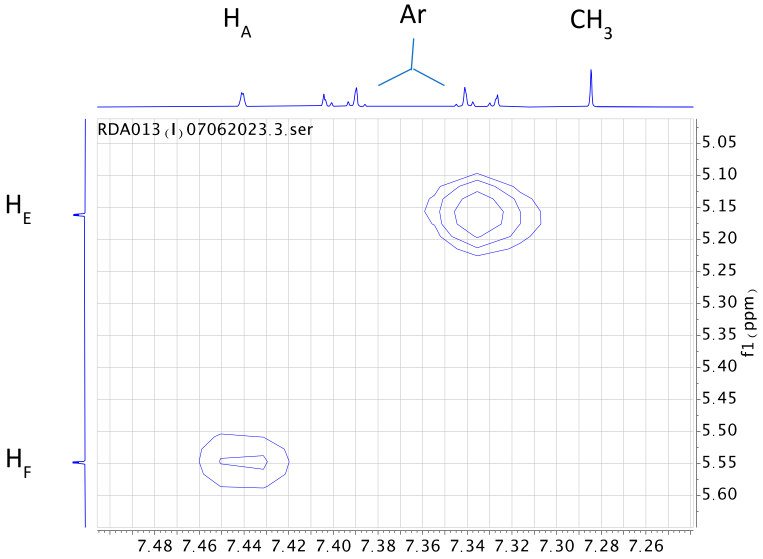

Figure S48. NOESY (600 MHz, CDCl3) correlations of compound **5n.**

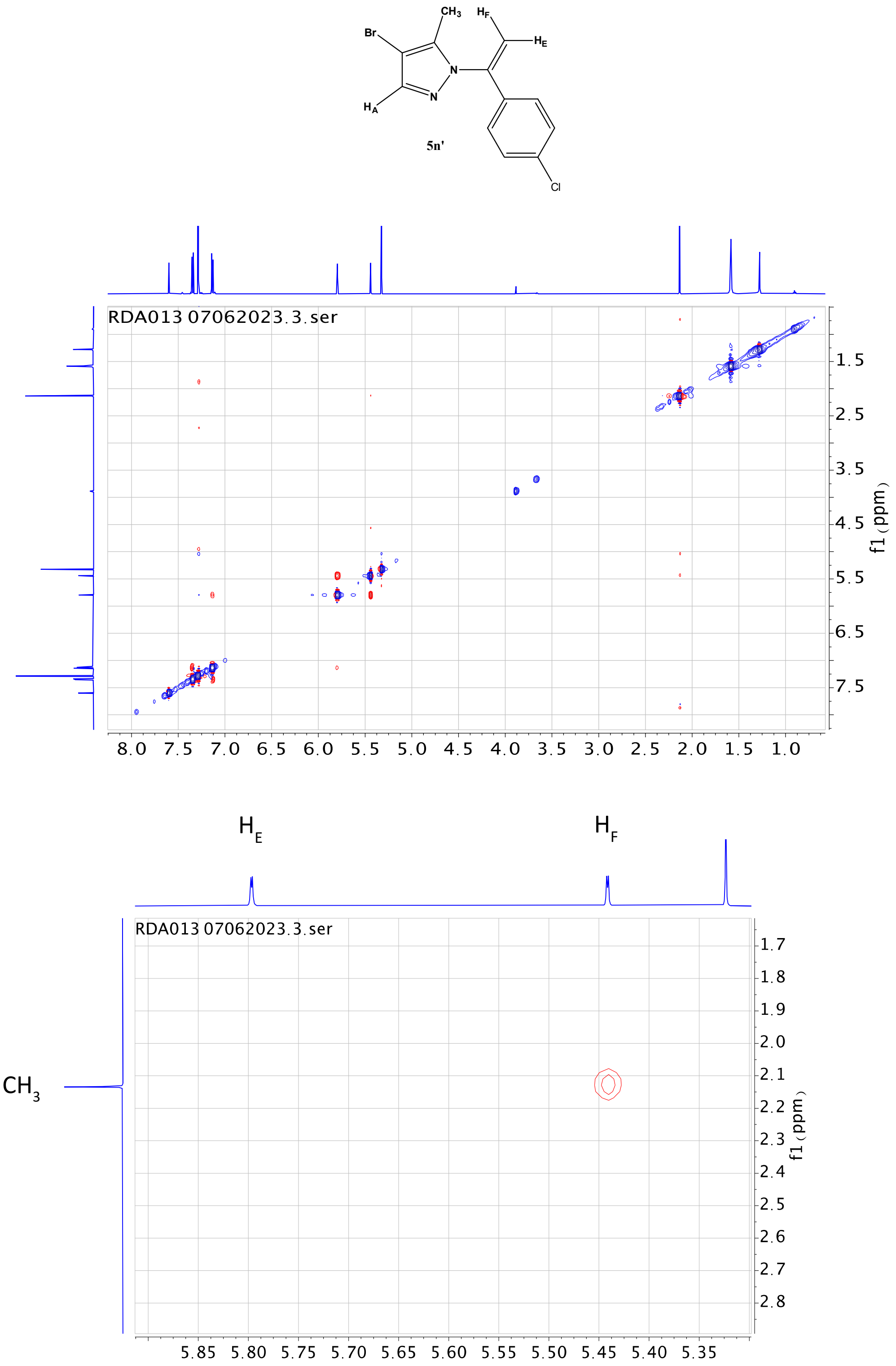

Figure S49. NOESY (600 MHz, $CDCl_3$) correlations of compound **5n′.**

References

9. Maki, Y.; Mori,H.; Endo, T. Xanthate-Mediated Controlled Radical Polymerization of N-Vinylindole Derivatives *Macromolecules* **2007**, *40*, 17, 6119-6130. https://doi.org/10.1021/ma062839q
14. Liao, Q.; Wang, Y.; Zhang, L., Xi, C. A General Copper- Catalyzed Coupling of azoles with Vinyl Bromides *J. Org. Chem.* **2009**, *74*, 6371-6373. https://doi.org/10.1021/jo901105r
16. Roche, M.; Frison, G.; Brion, J.-D.; Provot, O.; Hamze, A.; Alami, M. Csp^2–N Bond Formation via Ligand-Free Pd-Catalyzed Oxidative Coupling Reaction of *N*-Tosylhydrazones and Indole Derivatives. *J Org. Chem.* **2013**, *78*, 8485-8495. https://doi.org/10.1021/jo401217x
17. Zeng, X.; Cheng, G.; Shen, J.; Cui, X. Palladium-Catalyzed Oxidative Cross-Coupling of *N*-Tosylhydrazones with Indoles: Synthesis of *N*-Vinylindoles. *Org. Lett.* **2013**, *15*, 3022-3025. https://doi.org/10.1021/ol401217h
19. Rodygin, K., R., Bogachenkov, A., S.; Ananikov, V., P. Vinylation of a Secondary Amine Core with Calcium Carbide for Efficient Post-Modification and Access to Polymeric Materials *Molecules* **2018**, *23*, 648. https://doi.org/10.3390/molecules23030648
24. Nie, Z.; Lv, H; Li, H.; Su, M.; Yang, T.; Luo, W.; Liu, Q.; Guo, C. Synthesis of TerminalN-Vinylazoles fromAromatic Aldehydes, DMSO, and Azoles Based DMSO as Terminal Carbon Synthon *Adv. Synth. Catal.* **2021**, 363, 4621-4626. https://doi.org/10.1002/adsc.202100663.
30. Palomba, M.; Vinti, E.; Marini, F.; Santi, C.; Bagnoli, L. Synthesis of oxazino[4,3-*a*]indoles by domino addition-cyclization reactions of (1*H*-indol-2-yl)methanols and vinyl selenones in the presence of 18-crown-6. *Tetrahedron* **2016**, 72, 7059-7064. https://doi.org/10.1016/j.tet.2016.09.045
46. Tsuchimoto, T.; Aoki, K; Wagatsuma, T.; Suzuki, Y. Lewis Acid Catalyzed Addition of Pyrazoles to Alkynes: Selective Synthesis of Double and Single Addition Products *Eur. J. Org. Chem.* **2008**, 4035-4040. https://doi.org/10.1002/ejoc.200800353
